# *In situ* Raman spectroscopy reveals the structure evolution and lattice oxygen reaction pathway induced by the crystalline–amorphous heterojunction for water oxidation†

**DOI:** 10.1039/d2sc01043g

**Published:** 2022-04-22

**Authors:** Jianing Dong, Zhengxin Qian, Pan Xu, Mu-Fei Yue, Ru-Yu Zhou, Yanjie Wang, Zi-Ang Nan, Siying Huang, Quanfeng Dong, Jian-Feng Li, Feng Ru Fan, Zhong-Qun Tian

**Affiliations:** State Key Laboratory of Physical Chemistry of Solid Surfaces, College of Chemistry and Chemical Engineering, Innovation Laboratory for Sciences and Technologies of Energy Materials of Fujian Province (IKKEM), Xiamen University Xiamen 361005 China frfan@xmu.edu.cn; College of Optical and Electronic Technology, China Jiliang University Hangzhou China

## Abstract

One of the most successful approaches for balancing the high stability and activity of water oxidation in alkaline solutions is to use amorphous and crystalline heterostructures. However, due to the lack of direct evidence at the molecular level, the nano/micro processes of amorphous and crystalline heterostructure electrocatalysts, including self-reconstruction and reaction pathways, remain unknown. Herein, the Leidenfrost effect assisted electrospray approach combined with phase separation was used for the first time to create amorphous NiO_*x*_/crystalline α-Fe_2_O_3_ (a-NiO_*x*_/α-Fe_2_O_3_) nanowire arrays. The results of *in situ* Raman spectroscopy demonstrate that with the increase of the potential at the a-NiO_*x*_/α-Fe_2_O_3_ interface, a significant accumulation of OH can be observed. Combining with XAS spectra and DFT calculations, we believe that more OH adsorption on the Ni centers can facilitate Ni^2+^ deprotonation to achieve the high-valence oxidation of Ni^4+^ according to HSAB theory (Fe^3+^ serves as a strong Lewis acid). This result promotes the electrocatalysts to follow the lattice oxygen activation mechanism. This work, for the first time, offers direct spectroscopic evidence for deepening the fundamental understanding of the Lewis acid effect of Fe^3+^, and reveals the synergistic effect on water oxidation *via* the unique amorphous and crystalline heterostructures.

## Abstract 2

The amorphous NiO*_x_*/crystalline α-Fe_2_O_3_ heterojunctions were constructed and exhibited outstanding OER activities. Through the collaboration of multiple characterization techniques, the Lewis acid effect of Fe^3+^ was revealed at molecular level.

## Graphical abstract

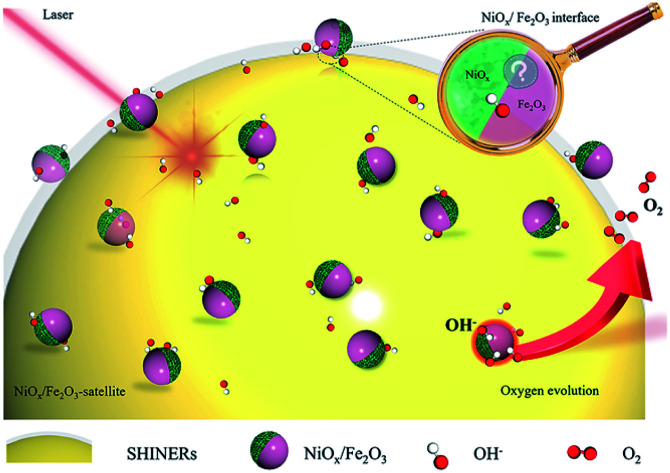

## Introduction

It is crucial to develop low-cost and efficient oxygen evolution reaction (OER) electrocatalysts, due to their key role in the anodic reaction of various electrocatalytic systems. However, since the OER is a four-electron transfer process and requires a large overpotential to surmount its sluggish reaction kinetics, the electrocatalytic performance is still unsatisfactory. Recently, Ni/Fe-based compounds are recognized as the most efficient electrocatalysts in alkaline media to replace the benchmark but expensive IrO_2_ and RuO_2_ for water oxidation. Furthermore, these electrocatalysts can be categorized as crystalline or amorphous materials.^1^ Usually, the amorphous electrocatalyst shows excellent OER activity, which can be explained by the following factors: excellent charge transfer, abundant defects/dangling bonds and coordinated unsaturated electronic structure.^2^ However, the active components are often thermodynamically unstable and leach out under OER conditions, resulting in the decrease of activity.^3^ To solve this issue, constructing a stable amorphous and crystalline boundary to balance and optimize its stability and activity is a potential method. Currently, the expected realization of the amorphous–crystalline boundary is mainly achieved through the shared edge/shared surface structure of amorphous and crystalline materials *via* sophisticated but complex designs.^1,4^ Therefore, how to easily and effectively construct crystalline and amorphous (named c–a) heterostructures is the main task of current research.

On the other hand, understanding the mechanism of the oxygen evolution reaction is crucial for the development of efficient OER electrocatalysts. Various mechanisms have been proposed to elucidate the OER process, including the conventional adsorbate evolution mechanism (AEM) and the lattice oxygen oxidation mechanism (LOM). The LOM of the OER involves direct O–O coupling that can bypass the highly relevant intermediate adsorption in the AEM, resulting in better OER activity.^5^ Therefore, clearly identifying the reaction pathway of the OER, especially at the molecular level, is essential for the design of electrocatalysts. Moreover, although the reaction mechanism of mixed Ni–Fe compounds has been extensively explored through diverse spectroscopic techniques (*e.g.*, Mössbauer, XAS and *in situ* Raman spectroscopy),^6–10^ the role of the Ni/Fe-based heterojunction for water oxidation, especially for the vigorous crystalline–amorphous heterojunction, is still in its infancy due to the lack of direct spectroscopic evidence, and the reaction pathway and structure evolution remain unclear. Moreover, it's well established that Fe^3+^ species was regarded as the Lewis acid center to promote the formation of Ni^4+^ species through *in situ* and *ex situ* X-ray absorption spectroscopy,^7,11^ whereas there was no direct evidence at the molecular level to elucidate this effect and detailed reaction process. Therefore, it is necessary to directly reveal the dynamic evolution and role of the heterojunction for the OER process at the molecular level under *in situ* reaction conditions.

In this study, we synthesized a-NiO_*x*_/α-Fe_2_O_3_ nanowire arrays *via* the Leidenfrost effect assisted electrospray method combined with subsequent phase separation. The a-NiO_*x*_/α-Fe_2_O_3_ nanowire arrays can realize complete self-restructuration evolution and exhibit excellent OER activity: *η*_500_ = 290 mV and long-time operation at ultra-high current densities of 1000 and 1200 mA cm^−2^ was performed, which is the urgent demand in alkaline electrolysis. Moreover, the self-reconstruction of electrocatalysts and water oxidation mechanisms were investigated using chemical probe studies, shell-isolated nanoparticle-enhanced Raman spectroscopy (SHINERS), XAS, electron paramagnetic resonance (EPR) spectroscopy and DFT calculations, and ultra-fast and complete self-reconstruction was achieved, which benefited from the c–a heterostructures and abundant oxygen defects. Furthermore, direct spectroscopic evidence shows that more OH has accumulated at the c–a interface, which cannot be observed in the sole electrocatalyst. This result elucidates the Lewis acid effect, that is, Fe^3+^ at the heterojunction acts as a strong Lewis acid to convert Ni^2+^ to Ni^4+^ through more OH adsorption, which alters the electrocatalysts to follow the lattice oxygen activation mechanism (LOM). This work discloses the synergistic effect of the crystalline–amorphous heterojunction on water oxidation.

## Results and discussion

### Synthesis and structural characterization of a-NiO_*x*_/α-Fe_2_O_3_ composites

**Fig. 1:**
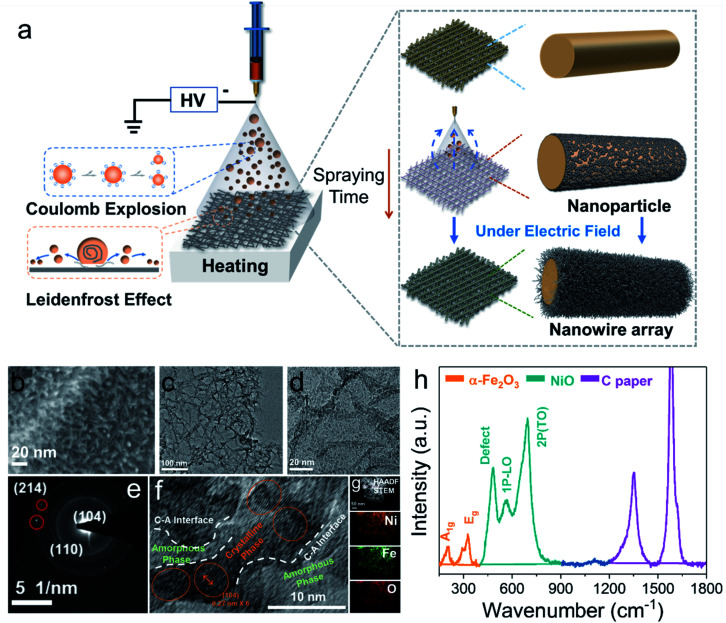
(a) Schematic illustration of the Leidenfrost effect assisted electrospray method. (b) SEM image of a-NiO_*x*_/α-Fe_2_O_3_ on carbon paper. (c) and (d) TEM images of a-NiO_*x*_/α-Fe_2_O_3_. SAED pattern (e), HRTEM image (f), and elemental maps (g) of a-NiO_*x*_/α-Fe_2_O_3_. (h) Raman spectra of a-NiO_*x*_/α-Fe_2_O_3_.

We first synthesized the a-NiO_*x*_/α-Fe_2_O_3_ nanowire arrays through the novel Leidenfrost effect assisted electrospray method. The nanowire arrays were prepared *via* the self-assembly method induced by an electric field (Fig. 1a). The detailed synthesis process can be obtained in the ESI† (Fig. S1–S5 and ESI Discussion†). The nanowire structure can fully contact the electrolyte, which provides a prerequisite for complete self-reconstruction.^12^ The morphology and structure of the electrocatalyst were characterized by scanning electron microscopy (SEM) and transmission electron microscopy (TEM). As shown in Fig. 1b, the SEM image shows the prepared uniform nanowire arrays. Furthermore, TEM revealed that the nanowires were assembled from many small nanoparticles under the action of an electric field (Fig. 1c and d). As shown in Fig. 1e, selected area electron diffraction (SAED) shows the nanocrystalline domains in the samples and exhibits (214), (110) and (104) crystal planes. It also indicates that the nanowires are assembled from nanoparticles. As shown in Fig. 1f, the c–a heterostructure can be clearly observed, and α-Fe_2_O_3_ nanoparticles were surrounded by a-NiO_*x*_ and the peak at 0.27 nm was attributed to the (104) plane of α-Fe_2_O_3_. More detailed structural information is shown in Fig. S7,† which is consistent with the SAED result. In addition, the EDX elemental mapping (Fig. 1g) of prepared samples shows a uniform heterojunction and the element distribution of Ni, Fe, and O.

To unravel the chemical composition and electronic properties of the electrocatalysts, X-ray photoelectron spectroscopy (XPS), X-ray diffraction (XRD), and Raman and EPR spectroscopy were performed. As shown in Fig. S7b,† the binding energies of Ni 2p_3/2_ and Ni 2p_1/2_ located at 855.6 eV and 873.8 eV could be ascribed to Ni^2+^, alongside shakeup satellites.^13^ In the high-resolution Fe 2p spectrum (Fig. S7c†), the two peaks with binding energies of 712.4 and 725.5 eV are assigned to Fe 2p_3/2_ and Fe 2p_1/2_ with two typical satellites, indicating the presence of Fe^3+^ belonging to Fe_2_O_3_.^14^ Fig. S7d† shows the magnified XPS spectrum of O 1s, the peaks of 529.6 eV, 531.4 eV and 533.4 eV representing the metal oxygen bond (O1) in the lattice, the oxygen species adsorbed by the oxygen vacancy (O2) and oxygen adsorbed on the surface (O3), respectively. The oxygen vacancy of the catalyst can be roughly estimated from the ratio of O2 to O1.^15^ It suggests that there are abundant oxygen vacancies in the electrocatalyst, which is beneficial for the rapid reconstruction of the electrocatalysts. The subsequent OER catalytic activity tests further verified that the catalyst with a Ni/Fe ratio of 3 : 1 (Ni_3_Fe_1_) presented the best performance. Therefore, we mainly focus on the catalysts with Ni_3_Fe_1_ in the following discussion, unless specified otherwise.

Furthermore, to obtain further evidence for structural deficiencies, EPR spectra of samples with different ratios were recorded. As shown in Fig. S7e,† the electrocatalyst with a Ni/Fe ratio of 3 : 1 shows the highest signal intensity compared with other Ni/Fe ratios with a *g* value of 2.003 (1 : 1 and 1 : 3, referred as Ni_1_Fe_1_ and Ni_1_Fe_3_, respectively), suggesting that there are more oxygen defects. Nevertheless, the coordination environment of the oxygen vacancy can exist in two kinds of defect coordination: Ni–O_v_ or Fe–O_v_. In order to clarify the coordination environment of oxygen vacancies, we analysed the structure of the electrocatalyst by Raman spectroscopy. As shown in Fig. 1h, the Raman peaks at 204 cm^−1^, 287 cm^−1^ and 326 cm^−1^ belong to the vibration peaks of A_1g_ and E_2g_ in α-Fe_2_O_3_, respectively.^16,17^ The peaks at 481 cm^−1^, 565 cm^−1^ and 692 cm^−1^ belong to the vibration peaks of NiO.^16,18^ Specifically, the Raman peak at 481 cm^−1^ represents the vibration peak of NiO defects, indicating that there are abundant oxygen defects in amorphous NiO. This result is also consistent with the EPR measurement. Therefore, we speculate that the coordination defect environment of oxygen vacancies exists in NiO rather than α-Fe_2_O_3_. XRD was used to analyse the crystal structure of the electrocatalysts. The electrocatalysts with different Ni/Fe ratios all exhibit the same diffraction peak of α-Fe_2_O_3_, whereas no obvious diffraction peak of NiO was observed (Fig. S7f†). Based on the above analysis, we conclude that the amorphous (NiO_*x*_)–crystalline (α-Fe_2_O_3_) heterojunction electrocatalysts with abundant oxygen vacancies have been successfully prepared through the Leidenfrost effect assisted electrospray method.

### Electrocatalytic properties of a-NiO_*x*_/α-Fe_2_O_3_ nanocomposites

**Fig. 2:**
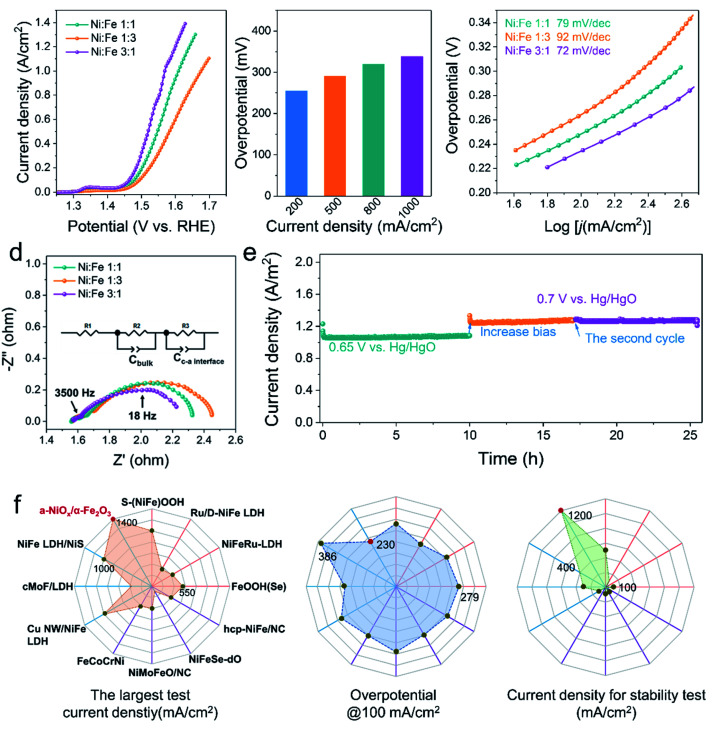
(a) LSV curves for OER measured on the different ratio samples at 5 mV s^−1^. (b) Overpotentials required to achieve current densities of 200, 500, 800 and 1000 mA cm^−2^ for the Ni_3_Fe_1_. (c) Tafel slopes derived from LSV curves. (d) EIS Nyquist plots of different ratio samples, measured at the 1.5 V *vs.* RHE. (e) Long-term stability tests at the constant potential. (f) Comprehensive comparisons of the OER performance of a-NiO_*x*_/α-Fe_2_O_3_ (Ni_3_Fe_1_) with those reported state-of-the-art catalysts in literature. Purple line: alloy/NiFe oxide; blue line: NiFe hydroxide; orange line: NiFe heterojunction electrocatalysts. From left to right: the largest test current; the overpotential @100 mA cm^−2^; current density for stability test.

Fig. 2a displays the linear sweep voltammogram (LSV) polarization curves of various electrocatalysts, clearly showing that our a-NiO_*x*_/α-Fe_2_O_3_ significantly outperforms the benchmark RuO_2_ catalyst (Fig. S8†). An overpotential of 260 mV is required for Ni_1_Fe_3_ to achieve a current density of 100 mA cm^−2^, which further decreases to 250 and 230 mV for Ni_1_Fe_1_ and Ni_3_Fe_1_ in 1 M KOH (pH ≈ 13.7), respectively. It is noteworthy that Ni_3_Fe_1_ only needs the overpotentials of 290 and 338 mV to achieve the large current densities of 500 and 1000 mA cm^−2^, respectively, exhibiting its superiority as a potential OER electrocatalyst candidate in practical applications (Fig. 2b). To probe reaction kinetics, the Tafel plots of various electrocatalysts were calculated, as depicted in Fig. 2c. The Tafel slope of Ni_1_Fe_3_ is determined to be 92 mV dec^−1^, which considerably decreases to 79 mV dec^−1^ and 72 mV dec^−1^ for Ni_1_Fe_1_ and Ni_3_Fe_1_, respectively. Moreover, EPR spectra of different Ni/Fe ratios have been recorded to investigate the effect of defects on the electrocatalysts (Fig. S7e†), and the peak intensity of oxygen defects gradually weakens as the content of NiO_*x*_ decreases. Therefore, more active sites may be there in amorphous NiO_*x*_ and the decreased Tafel slopes imply that oxygen defects can accelerate the reaction kinetics and the probable change of the rate-limiting step (RLS). To clarify the origin of the very high currents measured, we performed CV for double layer capacitance measurements to investigate the surface area effects of the different samples (Ni_3_Fe_1_, Ni_1_Fe_1_ and Ni_1_Fe_3_). As shown in Fig. S9,† after OER activation, the different samples exhibit different *C*_dl_ values. Ni_3_Fe_1_ shows the largest *C*_dl_ of about 10.88 mF cm^−2^, which indicates more active sites in Ni_3_Fe_1_ electrocatalysts. Moreover, Ni_1_Fe_1_ and Ni_1_Fe_3_ also exhibit a large *C*_dl_ of about 5.78 mF cm^−2^ and 3.61 mF cm^−2^, respectively. These large *C*_dl_ values can facilitate the realization of high currents.

Electrochemical impedance spectroscopy (EIS) is an important tool as it can be used to study the electrochemical behavior of the interface between the electrocatalyst and the electrolyte, and provides meaningful information regarding the composition. To understand the electrochemical behavior of a-NiO_*x*_/α-Fe_2_O_3_, an equivalent circuit was proposed (the inset of Fig. 2d). The EIS fitting results of Ni_3_Fe_1_ are shown in Fig. S10† and the corresponding element values are shown in Table S1.† The first semicircle in the high-frequency range (*e.g.* 3500 Hz) represents the space charge layer (*C*_bulk_), and the c–a interface components (*C*_c–a interface_ and R3) are associated with the impedance spectra for the low-frequency region (*e.g.* 18 Hz).^4,19^ The interfacial impedance dominates in charge transfer, which underscores the significant role of the c–a interface in boosting the OER activity. Notably, the Ni_3_Fe_1_ electrocatalyst shows the minimum interface resistance. Furthermore, the OER stability measurement was performed to test the stability performance of the c–a heterostructure for water oxidation. As shown in Fig. 2e, the electrocatalysts exhibit robust stability at large current densities of 1000 mA cm^−2^ and 1200 mA cm^−2^ for 25 h of continuous electrolysis, implying that the c–a heterojunction caused by phase separation is a promising way to endow electrocatalysts with excellent OER stabilities. In addition, as shown in Fig. 2f, the OER activity of Ni_3_Fe_1_ in terms of the largest test current density, overpotential at 100 mA cm^−2^ and stability test are superior to those of most reported and state-of-the-art NiFe-based OER electrocatalysts in 1 M KOH, more information can be obtained in Table S2.†

### Spectroscopy characterization and electrochemical technologies for exploring the electrocatalyst reconstruction

**Fig. 3:**
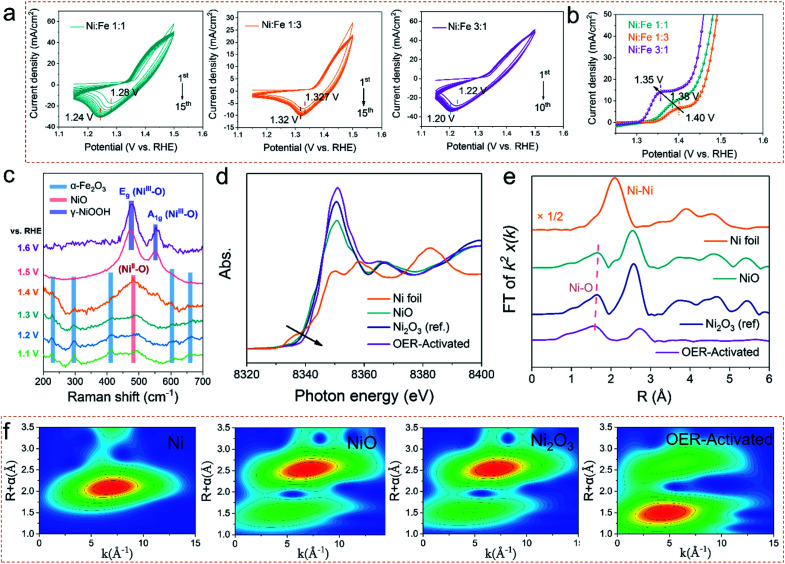
(a) The evolution of CV curves for Ni_1_Fe_1_, Ni_1_Fe_3_ and Ni_3_Fe_1_ in 1 M KOH at 100 mV s^−1^ between 1.15 and 1.5 V *vs.* RHE. (b) LSV curves in the electrooxidation range at 5 mV s^−1^. (c) *In situ* Raman spectroscopy measurements to investigate the self-reconstruction of Ni_3_Fe_1_. (d) Normalized nickel *K*-edge XANES spectra and EXAFS k2χ(k) Fourier transform (FT) spectra (e) of the OER-activated sample with Ni foil, NiO and Ni_2_O_3_ as references. (f) WT for the k2-weighted EXAFS signals of Ni foil, NiO, Ni_2_O_3_ and the OER-activated sample.

OER electrocatalysts usually undergo dynamic self-reconstruction under the conditions of the oxidation current, and the actual active catalytic species are formed *in situ* on the surface of the precatalysts.^20–22^ Therefore, the actual OER activity of the electrocatalyst is determined by the reaction rate and the extent of self-reconstruction.^23^ In order to deepen the understanding of the reconstruction reaction of the prepared catalysts, the electrochemical measurements and *in situ* electrochemical Raman spectroscopy were performed to evaluate the surface evolution. As shown in Fig. 3a, the electrocatalysts with different Ni/Fe ratios were activated by cyclic voltammetry. Compared with Ni_1_Fe_1_ and Ni_1_Fe_3_, Ni_3_Fe_1_ not only shows a faster reconstruction rate, but also needs only 10 cycles to realize the whole self-reconstruction process. At the same time, the potential of the reduction peak is further reduced from 0.32 V to 0.30 V. Correspondingly, the LSV curves (Fig. 3b) reveal that the Ni_3_Fe_1_ electrocatalyst also exhibits a lower oxidation potential of 1.35 V at a higher oxidation current density, suggesting faster reaction kinetics. It's crucial to understand the reasons for shifting of the Ni redox peak: the electronic effects (in the presence of Fe) or the concentration effect (nickel in the sample). We compare the LSV curves of different electrocatalysts. As shown in Fig. S11,† the oxidation peak of Ni shifts negatively with increasing NiFe ratio. However, Ni_3_Fe_1_ exhibits almost the same oxidation peak positions as a-NiO_*x*_ although the concentration of nickel in a-NiO_*x*_ is more than that of Ni_3_Fe_1_. This indicates that the electronic effects (the presence of Fe) can also shift the Ni redox peak to a lower potential. Therefore, we believe that both electronic effects and the concentration effect (nickel in the sample) can shift the Ni redox peak. Moreover, as the potential increases, the current density of a-NiO_*x*_ decreases, whereas that of the NiFe based electrocatalysts stays flat or even increases. This may be due to further oxidation of Ni^3+^ in NiFe based electrocatalysts. The results of electrochemical tests manifest that the electronic structure and different Ni contents of the catalysts can be effectively tuned by different concentrations of precursors and oxygen defects in the electrocatalysts.

To deeply investigate the degree of structural evolution and self-reconstruction of the catalyst, the evolution of surface oxygen species was monitored by *in situ* electrochemical Raman spectroscopy. As shown in Fig. 3c, the characteristic Raman signal at 482 cm^−1^–486 cm^−1^ can be recognized as the Ni^II^–O vibration in NiO_*x*_. The other Raman peaks can be ascribed to α-Fe_2_O_3_. In Raman spectroscopy, with the increase of the applied voltage, the intensity of the Raman peak of α-Fe_2_O_3_ continuously decreases, and the defect vibration peak of NiO constantly increases, indicating that the leaching out of α-Fe_2_O_3_ promotes the reconstruction of NiO. At the potentials of 600 mV and 700 mV, there are only two Raman peaks at 476 cm^−1^ and 552 cm^−1^, which represent *E*_g_ bending vibration (*δ*(Ni–O)) and A_1g_ stretching vibration (*ν* (Ni–O)) modes in nickel oxyhydroxide (NiOOH), respectively.^24^ FeOOH cannot be observed during the *in situ* monitoring process, although it can be detected by *ex situ* characterization due to anodic deposition. Therefore, we believe that the main active species is NiOOH during the OER process. And Fe compounds may perform the supporting role to promote the Ni-based electrocatalysts to transfer the real electrocatalysts. The variation of *δ*(Ni–O)-to-*ν*(Ni–O) ratios (labelled to *I*_*δ*/*ν*_) can be used to distinguish the crystal phases formed by NiOOH. Generally, the intensity of *ν*(Ni–O) is relatively lower (leading to higher *I*_*d*/ν_) in γ-NiOOH due to its looser and more disordered structure.^25,26^ Therefore, such a higher *I*_*δ*/*ν*_ in the prepared electrocatalyst manifests the direct formation of the γ-NiOOH structure when surface oxidation occurs. It is regarded that highly oxidized Ni^4+^ species exists in γ-NiOOH due to the statistical Ni value higher than + 3.7, which is beneficial for the lattice oxygen activation mechanism (LOM) reaction.^27^ Moreover, we used SEM and TEM to investigate the reconstructed electrocatalyst to reveal the degree of reconstruction. As shown in Fig. S12,† the nanowire arrays are completely converted to the nanosheets. The TEM images show that the active electrocatalyst species is NiOOH/FeOOH, which is also confirmed by the normal Raman and XPS measurements (Fig. S13a–d†). Notably, there are no Raman peaks of pristine NiO and α-Fe_2_O_3_ at high potentials, and the nanowire arrays are all converted into nanosheets. Therefore, we infer that the whole electrocatalyst has been completely reconstructed, thus exhibiting better OER catalytic performance. Moreover, as shown in Fig. S13b,† the peak intensity of M–O in the O 1s spectrum is higher than that of the pristine sample, indicating that the content of lattice oxygen increases after the OER process, suggesting a potential LOM reaction pathway.^28^

*Ex situ* Ni *K*-edge XAS spectra measurements were employed to investigate the subtle changes in the oxidation states and the local bonding environments throughout the nanocomposite samples. According to the relative absorption edge positions in the Ni *K*-edge X-ray absorption near-edge spectra (Fig. 3d), and commercial Ni, NiO, and Ni_2_O_3_ samples used as references, the shift of the absorption threshold to a higher energy for the OER-activated sample suggests that higher valence nickel sites are formed.^29^ Here, the integral method is used to quantify the valence state of nickel (Fig. S14†). The result (Fig. S14†) shows that the Ni valence state increases to +3.8 after the OER activation. Moreover, the Ni–O bond length is then probed by extended X-ray absorption fine structure (EXAFS). It is clear that as the valence state of nickel increases, the average Ni–O bond length decreases, which indicates that the orbital hybridization between Ni-3d and O-2p increases (Fig. 3e).^29^ Furthermore, the wavelet-transform (WT) contour plots (Fig. 3f) were investigated to provide an intuitive method to further determine the coordination environment of Ni atoms and the relative intensity and the distance of the Ni–O coordination was found to increase and decrease in the order of OER-activated – Ni_2_O_3_–NiO. Considering the results of *in situ* Raman and XAS spectra, we conclude that the dynamically formed Ni^4+^ species in γ-NiOOH serves as a veritably active site for water oxidation after the OER activation.

### Chemical probe studies, *in situ* Raman studies and DFT calculations towards the OER reaction mechanisms

**Fig. 4:**
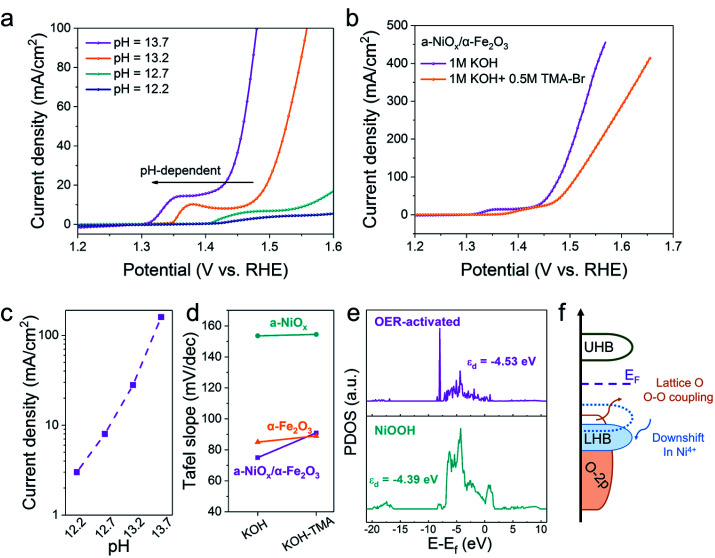
(a) pH dependence of the OER activities of a-NiO_*x*_/α-Fe_2_O_3_ at a scan rate of 5 mV s^−1^ (b) LSV curves of a-NiO_*x*_/α-Fe_2_O_3_ in 1 M KOH and 1 M KOH with TMA+ at 5 mV s^−1^. (c) Current densities of a-NiO_*x*_/α-Fe_2_O_3_ as a function of the pH value. (d) Tafel slopes of a-NiO_*x*_, α-Fe_2_O_3_ and a-NiO_*x*_/α-Fe_2_O_3_ in 1 M KOH and 1 M KOH with TMA^+^, respectively. (e) PDOS of Ni 3d orbitals in the OER-activated sample and NiOOH. (f) Schematic illustration of activating lattice oxygen induced by Ni^4+^ species in the Mott–Hubbard model.

The OER reaction pathway on oxides through the evolution of lattice oxygen typically exhibits pH-dependent activity. To clarify the reaction pathway of a-NiO_*x*_/α-Fe_2_O_3_, we performed the chemical probe studies of the OER in various pH and the tetramethylammonium cation (TMA^+^) solutions.^28–30^ As shown in Fig. 4a and 4c, the OER performance of a-NiO_*x*_/α-Fe_2_O_3_ is highly correlated with pH because the deprotonation process is easier at higher pH and the onset potential decreases as the pH of the solution increases.^30–32^ Therefore, a-NiO_*x*_/α-Fe_2_O_3_ exhibits strong pH-dependence, following the potential LOM mechanism. Furthermore, TMA+ was introduced into the electrolyte to capture the peroxided species (O_2_^2−^/O_2_^−^) produced from the O–O coupling, which is distinguished from AEM.^30,33^ As shown in Fig. 4b, S15a and b,† the OER activity of a-NiO_*x*_/α-Fe_2_O_3_ significantly reduced compared with that of a-NiO_*x*_ and α-Fe_2_O_3_ in TMA cationic solutions. The Tafel slope increased from 75 mV dec^−1^ to 91 mV dec^−1^ due to the strong suppression of the LOM by TMA^+^, while there was no obvious Tafel slope change in a-NiO_*x*_ and α-Fe_2_O_3_ (Fig. 4d). This result indicates that the crystalline–amorphous heterojunction can change the OER pathway and realize the high OER activity of a-NiO_*x*_/α-Fe_2_O_3_. Moreover, the Mott–Hubbard splitting can be used to describe the d-orbital splitting of the late translation metals owing to the strong d–d onsite Coulomb interaction: electron-filled lower Hubbard band (LHB) and empty upper Hubbard band (UHB).^34^ As shown in Fig. 4e, the partial density of states (PDOS) of the Ni 3d orbitals in different electrocatalysts were calculated. The Ni d-band center of the OER-activated sample is −4.53 eV, which is 0.14 eV lower than that of NiOOH. The highly oxidized Ni^4+^ species downshift the LHB to probably penetrate p-band of oxygen ligands as shown in Fig. 4f. This transition from a Mott–Hubbard insulator to a charge-transfer insulator can introduce lattice O to form O–O coupling.^27^

Several studies have reported that Fe impurities in the electrolyte will affect the OER performance.^35,36^ To exclude the influence of Fe impurities, CV measurements of electrocatalysts and controlled experiment were employed. Firstly, as shown in Fig. 3a, the redox peaks shift to lower potentials of different NiFe ratio electrocatalysts. These results are contrary to those reported in Boettcher's work. In his work,^35^ the redox peaks shift to higher potentials in the presence of Fe impurities. Therefore, we believe that the Fe species in heterostructures propose a more pronounced effect on OER performance and the effect of Fe impurities may be negligible due to their very low content. Secondly, the electrochemical performance of pure a-NiO_*x*_ synthesized with the same method was performed as a reference (Fig. S16†). We believe that we can obtain the specific role of Fe in heterojunctions by comparing with a-NiO_*x*_ to exclude the interference of Fe impurities.

**Fig. 5:**
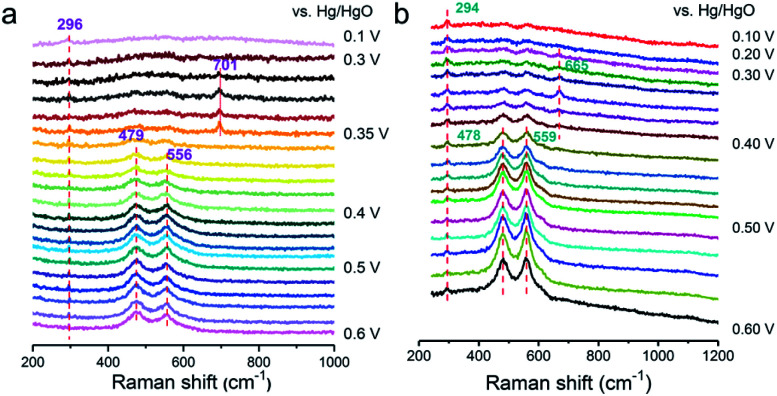
*In situ* SHINER spectra of the OER on a-NiO_*x*_/α-Fe_2_O_3_ in 1  M KOH at different potentials dissolved in H_2_O (a) and D_2_O (b).

To deeply investigate the origin of the reaction mechanism change, the electrochemical SHINERS-satellite structure was employed to examine the OER performance of a-NiO_*x*_/α-Fe_2_O_3_ and NiO_*x*_ electrocatalysts. As shown in the TEM image in Fig. S17,† a-NiO_*x*_/α-Fe_2_O_3_ is uniformly dispersed on the surface of the SHINs due to the electrostatic interaction. Then combined with *in situ* electrochemical enhanced Raman technology, the experiment was performed in 1 M KOH from 0.6 V to 0.1 V *versus* Hg/HgO (Fig. 5a). The characteristic peak at 296 cm^−1^ is attributed to the Fe–O bond. Obviously, 0.41 V–0.5 V (Vs. Hg/HgO) exhibits two pairs of characteristic peaks at 479 cm^−1^ and 556 cm^−1^, corresponding to the vibration mode of Ni–O in NiOOH.^28^ Special attention is paid to the apparent peak value of 701 cm^−1^ in the range of 0.35 V to 0.4 V. According to previous literature reports, the Raman frequency of the adsorbed OH with rocking mode has been observed at 778 cm^−1^ on PtNi, the bending mode at 790 cm^−1^ on Au and 716 cm^−1^ on RuO_2_·*x*H_2_O.^37–39^ Therefore, it is speculated that it may be consistent with the intermediate species of *OH. The deuterium isotope experiment of NiO_*x*_/Fe_2_O_3_-SAT was conducted to verify this hypothesis (Fig. 5b). The peak at 701 cm^−1^ is red-shifted to a lower wave number around 665 cm^−1^, which means that this species is related to the H atom. The DFT calculated structural model (Fig. S18†) suggests that the optimized vibration frequency of *OH absorbed on the Ni site is 685 cm^−1^, which is consistent with the *in situ* Raman results. In addition, compared with the *in situ* electrochemical Raman spectrum of NiO_*x*_-SAT (Fig. S19†), no peak response of *OH was observed. Moreover, recent studies have shown that Fe^3+^ species are regarded as Lewis acid centers to promote the formation of Ni^4+^ species.^7^ In the HSAB theory, the Fe^3+^ center is a hard acid that has weak bonding with Lewis soft base (nearly neutral OH_ad_).^40,41^ The unbalanced binding energy may drive OH_ad_ towards the Ni^3+^ center to deliver further oxidation. It is known that Ni favors the adsorption of OH_ad_ species.^42^ Therefore, it is speculated that the formed a-NiO_*x*_/α-Fe_2_O_3_ interface enhances the ability of capturing OH in the solution, thereby changing the reaction mechanism, and the adsorption of OH is a relatively important step in the oxygen evolution reaction of the LOM pathway under alkaline conditions.

**Fig. 6:**
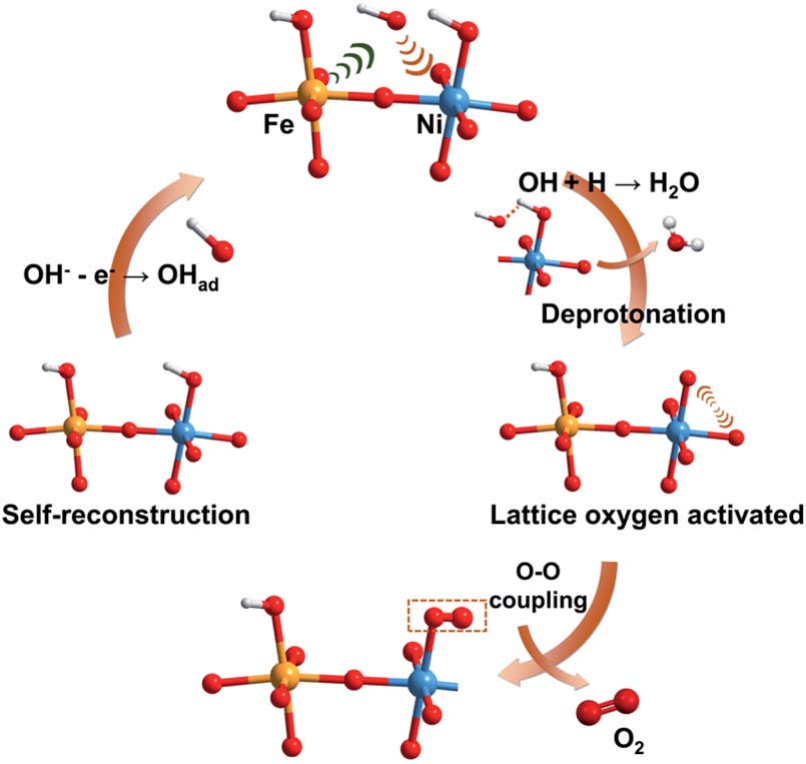
Schematic illustration of the proposed overall OER pathway for the self-reconstruction electrocatalyst. The yellow, blue, red and white balls represent Fe, Ni, O and H atoms, respectively.

By integrating experimental evidence with the simulated results, we present an overall depiction of water oxidation on the self-reconstruction electrocatalyst (FeOOH/NiOOH model) to justify the improved OER activity of the corresponding a-NiO_*x*_/α-Fe_2_O_3_, as schemed in Fig. 6. The electrical modification of the Fe–O–Ni moiety promotes the formation of Ni^4+^ species. Primarily, the pre-adsorbed OH_ad_ species accumulated at the Ni site is driven by Fe^3+^ as a Lewis acid, realizing the deprotonation step. This process allows for the direct coupling of the O intermediate and activated lattice oxygen, which is favorable for high-valence metal cations in terms of energy.^43,44^ The formed OO species can therefore be considered as a newly produced O_2_ molecule that will undergo subsequent oxygen evolution.^5^ As a result, benefiting from the accumulated OH_ad_ by Fe^3+^ and dynamically constructed Ni^4+^ species, the a-NiO_*x*_/α-Fe_2_O_3_ electrocatalyst exhibits ultra-low overpotential and excellent OER activity.

## Experimental

### Synthesis of amorphous NiO_*x*_/crystalline α-Fe_2_O_3_, NiO_*x*_ and α-Fe_2_O_3_ by the Leidenfrost effect assisted electrospray method

First, 1 × 0.5 cm^2^ carbon paper is cleaned by ultrasonication in acetone, ethanol and DI water for 10 min, respectively and dried under ambient conditions. Then, the carbon paper was placed on the copper heating plates and heated up to 290 °C to accomplish the Leidenfrost effect for water droplets. 0.1 M FeCl_3_·6H_2_O and 0.1 M NiCl_3_·6H_2_O were dissolved in DI water, and different volume ratios of Ni to Fe (1 : 1, 1 : 3 and 3 : 1) were mixed as precursors. Then the solution was injected into a fused silica capillary (50 μm inner diameter and 150 μm outer diameter) by using a 500 μL syringe (Hamilton) and a syringe pump (Harvard apparatus) at a rate of 20 μL min^−1^ for 10 min. 3 ± 0.5 kV is applied on the needle to form a Taylor-cone mode with a distance of 3 cm between the needle and substrate. The synthesis procedures of NiO_*x*_ and α-Fe_2_O_3_ are the same as above, except that no additional FeCl_3_ or NiCl_2_ is added. The mass loadings of different NiFe ratio electrocatalysts in this work are all about 2.3 mg cm^−2^.

### Synthesis of RuO_2_/NF

RuO_2_ catalysts are loaded on the carbon paper to fabricate RuO_2_/NF. Briefly, a homogeneous ink was obtained by dispersing the commercial 7 mg RuO_2_ catalysts in the mixture of 200 μL water and 5 μL Nafion by ultrasonic treatment. And then, all the ink was dropped on the carbon paper to ensure that the loading mass of RuO_2_ was 2.5 mg cm^−2^.

### Preparation of 120 nm Au@2 nm SiO_2_ nanoparticles (SHINs)

According to the literature,^45^ 120 nm Au core nanoparticles were prepared by the seed-mediated growth method. First, 200 mL of 0.01 wt% HAuCl_4_ aqueous solution is heated to boiling, and quickly injected 2 mL of 1 wt% sodium citrate aqueous solution for 40 minutes to obtain 45 nm Au. Then 3 mL of 45 nm Au in the ice bath was taken as seeds, 0.4 mL of 0.1 wt% ascorbic acid and 0.1 mL of 0.1 wt% sodium citrate were added to Au seed solution and stirred for 10 minutes. Then 0.7 mL of 0.024 M HAuCl_4_·4H_2_O solution was added and reacted for 30 minutes to obtain 120 nm Au. The SHINs were synthesized according to the previous publications of our group (1). 0.4 mL of 1 mM APTMS solution was added to 20 mL of 120 nm Au solution and stirred for 15 minutes at room temperature. Then 3.2 mL of 0.54 wt% sodium silicate solution was added to the solution and the pH was adjusted to about 10. Finally, the solution was stirred overnight to obtain silica-coated 120 nm Au with a shell layer of 2 nm thickness (SHINs). The obtained sample was centrifuged and washed three times with ultrapure water.

### Self-assembly of NiO_*x*_/Fe_2_O_3_ on SHINs (NiO_*x*_/Fe_2_O_3_-SAT)

NiO_*x*_/Fe_2_O_3_ on the SHIN satellite structure is self-assembled through the electrostatic interaction. A piece of the carbon cloth sample was taken, 3 mL ultrapure water was added and sonicated for 30 minutes to obtain a suspension. Then 1 mL of the SHIN solution prepared above was added, and the satellite structured NiO_*x*_/Fe_2_O_3_ (NiO_*x*_/Fe_2_O_3_-SAT) was obtained after 30 minutes of sonication in the ice region. Finally, it was cleaned three times with ultrapure water by centrifugation.

### Raman measurements

Raman experiments were carried out with a confocal microscope Raman system Xplora (Jobin-Yvon France). The excitation wavelength was 638 nm from a He–Ne laser, and a 50× microscope objective with a 0.55 numerical aperture was used for all Raman measurements. Wavenumber calibration was regularly verified by acquiring the Raman peak at 520 cm^−1^ of a silicon crystal. *In situ* electrochemical Raman experiments were performed in a homemade Raman cell with a NiO_*x*_/Fe_2_O_3_-SHIN decorated glassy carbon electrode as the working electrode, a Pt wire as the counter electrode, and a Hg/HgO electrode (filled with 1 M KOH) as the reference electrode. An Autolab PGSTAT30 (Metrohm) potentiostat was used to control the potential. To avoid the influence of electro-oxidation, potential negative sweep is used to explore the oxygen evolution process, and the potential is from 600 mV to 100 mV (*vs.* Hg/HgO).

### Materials characterization

The morphology of electrocatalysts was observed using field emission scanning electron microscopy (FE-SEM, Hitachi S-4800, Zeiss-Gmini500) and transmission electron microscopy (TEM, FEI Tecnai F-30 operated at 200 kV). In addition, high-resolution transmission electron microscopy (HRTEM) with energy dispersive X-ray (EDX) spectroscopy analysis (TEM, FEI Tecnai F-30 operated at 200 kV) was employed to give more detailed morphologies. The valence state, surface chemical state, and compositions were explored by X-ray photoelectron spectroscopy (XPS, Thermo Fisher Scientific K-Alpha). XRD patterns were analyzed on an X-ray diffractometer (Rigaku, RINT2500) with a Cu Kα radiation source. EPR measurement was performed by using a Bruker EMX-10/12 under 100 K conditions.

The X-ray absorption spectra at the Ni *K*-edge were recorded at the XAS station (BL14W1) of the Shanghai Synchrotron Radiation Facility (SSRF). The sample preparation procedure for XAS investigations is as follows: the Ni foil sample was commercially available at the XAS station. The NiO and Ni_2_O_3_ powder were prepared into 13 mm diameter discs by tableting. Then, these discs were encapsulated with Kapton tape for XAS testing. The OER-activated sample was directly encapsulated with Kapton tape after activation. The electron storage ring was operated at 3.5 GeV. A Si (311) double-crystal was used as the monochromator, and the data were collected using a solid-state detector. The X-ray absorption of Ni foil at the Ni *K*-edge was measured for energy calibration and data processing as a standard sample. The obtained XAS original data were processed for the background, pre-edge line, post-edge line correction and normalized in Athena. A *k* range of 2–11 Å^−1^ and *k*-weight of 2 were used for all the Ni-samples.

### Electrocatalytic performance measurement

The water oxidation measurements were performed through an electrochemical work station (CHI 660E, Shanghai Chenhua) with a typical three-electrode system: different electrocatalysts as working electrodes, a graphite rod as the counter electrode and mercury oxide (Hg/HgO) as the reference electrode, and 1 M KOH solution as the alkaline electrolyte. The quartz beaker was used as the electrochemical cell and we cleaned the cell with H_2_SO_4_ and then rinsed with deionized water (18.2 MΩ cm) before each experiment. To avoid potential capillary action, which allows possible catalysis of the conductive electrode clip (Pt), the electrocatalysts partially immerse in solution. In this case, the real working electrochemical active area in solution is 0.3 cm^2^. Current densities are normalized with the electrode geometric surface areas. The pH of different KOH solutions was determined by using a pH meter. The uncompensated resistance change for the different KOH concentrations is shown in Table S3.† The OER polarization curves were measured at a scan rate of 5 mV s^−1^. All potentials reported were calibrated to that of the reversible hydrogen electrode (RHE) by *E*_RHE_ = *E*_SCE_ + 0.098 V + 0.059 × pH, and LSV curves were corrected for an ohmic drop (85% for electrochemical analysis in Fig. 3 and 4 and 90% for large current experiments in Fig. 2) to evaluate the true activity of the electrocatalysts. Electrochemical impedance spectroscopy was performed in a 1 M KOH solution at 1.5 V (*vs.* RHE) from 10^5^–10^−1^ Hz with an AC voltage amplitude of 5 mV to measure the system resistance. All the presented curves were in their steady-state after several cycles.

### Computational method

All the density functional theory (DFT) calculations were performed on the Vienna *ab initio* Simulation Package (VASP).^46,47^ The exchange–correlation interactions were described by generalized gradient approximation in the form of the Perdew–Burke–Emzerh functional (GGA + PBE).^48^ The ion cores and valence electron interactions were treated by expanding a plane wave basis set with a kinetic energy cutoff of 400 eV, using a projector augmented-wave (PAW) method.^49^ The spin-polarization was considered in all calculations. The on-site coulombic interaction based on DFT + U methods reported by Dudarev *et al.* was adopted to describe the strong localized d orbital of transition metal. In the present work, a value of *U* = 6.6 eV and 3.5 eV was applied to Ni 3d and Fe 3d orbitals according to previous reports.^50^ In addition, van der Waals interactions were corrected by using the DFT-D3 approach.^51,52^ The Brillouin zone was sampled by using a 4 × 5 × 2 and 4 × 2 × 3 Monkhorst–Pack^53^*k*-point mesh for NiOOH and FeOOH bulk geometry optimization, respectively. And a 1 × 1 × 1 Gamma-centered Monkhorst–Pack *k*-point grid was used for all slab model calculations. In addition, a denser 4 × 4 × 1 *K*-point mesh was selected for calculating the electron density. The heterostructure FeOOH (010)/NiOOH (122) was constructed by five layers of NiOOH (122) and 3 layers of FeOOH(010)-p(2 × 2). To simulate the oxygen evolution reaction (OER) occurring at the heterostructure interface, half of the FeOOH (010) layer surface was removed. A vacuum thickness of 15 Å was applied to avoid the periodic image interaction. The bottom four layers were fixed at their bulk position and the top four layers and adsorbate was allowed to relax until the total energy and force were lower than 10^−4^ eV and 0.05 eV Å^−1^, respectively.

## Conclusions

In summary, we for the first time, provide a unique synthesis method to construct a-NiO_*x*_/α-Fe_2_O_3_ heterojunctions *via* combining the Leidenfrost effect aided electrospray approach with phase separation. Furthermore, *in situ* Raman spectroscopy, chemical probe studies and other spectral technologies are employed to explore the structure evolution of the electrocatalyst and reaction pathway for water oxidation at the c–a heterojunction. a-NiO_*x*_/α-Fe_2_O_3_ undergoes ultra-fast reconstruction and exhibits state-of-art OER activities (*η*_500_ = 290 mV). Furthermore, *in situ* SHINER spectra show that more OH is accumulated at the c–a interface. Chemical probe studies and XAS spectra reveal that this accumulated OH could promote Ni^4+^ species formation and alter the reaction pathway to follow the lattice oxygen mechanism to deliver faster reaction kinetics. This work is an important step toward understanding the synergistic effect of the crystalline–amorphous heterojunction for water oxidation and even in other electrocatalytic reactions.

## Data availability

The data supporting the findings of this study are available within the article and in the ESI.†

## Author contributions

F. R. Fan and J. N. Dong conceived the idea, designed the experiments and analyze the data. P. Xu helped characterize and analysis the structure of samples under the supervision of Q. F. Dong. M.-F. Yue conducted the theoretical calculation, Z. X. Qian and R.-Y. Zhou conducted the Raman experiment under the supervision of J.-F. Li. Y. J. Wang, Z.-A. Nan, S. Y. Huang and J. N. Dong conducted most experiments under the supervision of F. R. Fan and Z.-Q. Tian. Z.-Q. Tian provided scientific guidance throughout the study. J. N. Dong and Z. X. Qian wrote the manuscript with the contribution of all authors.

## Conflicts of interest

The authors declare no competing financial interest.

## Supplementary Material

SC-013-D2SC01043G-s001

## References

[cit1] Guan D., Zhou W., Shao Z. (2021). Small Sci..

[cit2] Zhai Y., Ren X., Yan J., Liu S. (2020). Small Struct..

[cit3] Feng C., Wang F., Liu Z., Nakabayashi M., Xiao Y., Zeng Q., Fu J., Wu Q., Cui C., Han Y., Shibata N., Domen K., Sharp I. D., Li Y. (2021). Nat. Commun..

[cit4] Han H., Choi H., Mhin S., Hong Y. R., Kim K. M., Kwon J., Ali G., Chung K. Y., Je M., Umh H. N., Lim D. H., Davey K., Qiao S. Z., Paik U., Song T. (2019). Energy Environ. Sci..

[cit5] Zhang N., Chai Y. (2021). Energy Environ. Sci..

[cit6] Chen J. Y., Dang L., Liang H., Bi W., Gerken J. B., Jin S., Alp E. E., Stahl S. S. (2015). J. Am. Chem. Soc..

[cit7] Li N., Bediako D. K., Hadt R. G., Hayes D., Kempa T. J., von Cube F., Bell D. C., Chen L. X., Nocera D. G. (2017). Proc. Natl. Acad. Sci. U. S. A..

[cit8] Bai L., Lee S., Hu X. (2021). Angew. Chem., Int. Ed. Engl..

[cit9] Hu C., Hu Y., Fan C., Yang L., Zhang Y., Li H., Xie W. (2021). Angew. Chem., Int. Ed. Engl..

[cit10] Trotochaud L., Young S. L., Ranney J. K., Boettcher S. W. (2014). J. Am. Chem. Soc..

[cit11] Li N., Keane T. P., Veroneau S. S., Hadt R. G., Hayes D., Chen L. X., Nocera D. G. (2020). Proc. Natl. Acad. Sci. U. S. A..

[cit12] Liu X., Meng J., Ni K., Guo R., Xia F., Xie J., Li X., Wen B., Wu P., Li M., Wu J., Wu X., Mai L., Zhao D. (2020). Cell Rep. Phys. Sci..

[cit13] Wang Q., Huang X., Zhao Z. L., Wang M., Xiang B., Li J., Feng Z., Xu H., Gu M. (2020). J. Am. Chem. Soc..

[cit14] Jiang Y., Liao J. F., Chen H. Y., Zhang H. H., Li J. Y., Wang X. D., Kuang D. B. (2020). Chem.

[cit15] Zhao Z., Shao Q., Xue J., Huang B., Niu Z., Gu H., Huang X., Lang J. (2022). Nano Res..

[cit16] Bhushan M., Kumar Y., Periyasamy L., Viswanath A. K. (2019). Nanotechnology.

[cit17] Ahmmad B., Leonard K., Shariful Islam M., Kurawaki J., Muruganandham M., Ohkubo T., Kuroda Y. (2013). Adv. Powder Technol..

[cit18] Radinger H., Connor P., Tengeler S., Stark R. W., Jaegermann W., Kaiser B. (2021). Chem. Mater..

[cit19] Anderson N. C., Carroll G. M., Pekarek R. T., Christensen S. T., van de Lagemaat J., Neale N. R. (2017). J. Phys. Chem. Lett..

[cit20] Liu X., Guo R., Ni K., Xia F., Niu C., Wen B., Meng J., Wu P., Wu J., Wu X., Mai L. (2020). Adv. Mater..

[cit21] Wang J., Kim S. J., Liu J., Gao Y., Choi S., Han J., Shin H., Jo S., Kim J., Ciucci F., Kim H., Li Q., Yang W., Long X., Yang S., Cho S. P., Chae K. H., Kim M. G., Kim H., Lim J. (2021). Nat. Catal..

[cit22] Dionigi F., Zeng Z., Sinev I., Merzdorf T., Deshpande S., Lopez M. B., Kunze S., Zegkinoglou I., Sarodnik H., Fan D., Bergmann A., Drnec J., Araujo J. F., Gliech M., Teschner D., Zhu J., Li W. X., Greeley J., Cuenya B. R., Strasser P. (2020). Nat. Commun..

[cit23] Li S., Li Z., Ma R., Gao C., Liu L., Hu L., Zhu J., Sun T., Tang Y., Liu D., Wang J. (2021). Angew. Chem., Int. Ed. Engl..

[cit24] Wang Y., Zhu Y., Zhao S., She S., Zhang F., Chen Y., Williams T., Gengenbach T., Zu L., Mao H., Zhou W., Shao Z., Wang H., Tang J., Zhao D., Selomulya C. (2020). Matter.

[cit25] Louie M. W., Bell A. T. (2013). J. Am. Chem. Soc..

[cit26] Huang J., Li Y., Zhang Y., Rao G., Wu C., Hu Y., Wang X., Lu R., Li Y., Xiong J. (2019). Angew. Chem., Int. Ed. Engl..

[cit27] Zhang N., Feng X., Rao D., Deng X., Cai L., Qiu B., Long R., Xiong Y., Lu Y., Chai Y. (2020). Nat. Commun..

[cit28] Ren X., Wei C., Sun Y., Liu X., Meng F., Meng X., Sun S., Xi S., Du Y., Bi Z., Shang G., Fisher A. C., Gu L., Xu Z. J. (2020). Adv. Mater..

[cit29] Zhang L., Wang L., Lin H., Liu Y., Ye J., Wen Y., Chen A., Wang L., Ni F., Zhou Z., Sun S., Li Y., Zhang B., Peng H. (2019). Angew. Chem., Int. Ed. Engl..

[cit30] Huang Z. F., Song J., Du Y., Xi S., Dou S., Nsanzimana J. M. V., Wang C., Xu Z. J., Wang X. (2019). Nat. Energy.

[cit31] Huang Z. F., Xi S., Song J., Dou S., Li X., Du Y., Diao C., Xu Z. J., Wang X. (2021). Nat. Commun..

[cit32] Diaz-Morales O., Ferrus-Suspedra D., Koper M. T. M. (2016). Chem. Sci..

[cit33] Li X., Xiao L., Zhou L., Xu Q., Weng J., Xu J., Liu B. (2020). Angew. Chem., Int. Ed. Engl..

[cit34] Imada M., Fujimori A., Tokura Y. (1998). Rev. Mod. Phys..

[cit35] Trotochaud L., Young S. L., Ranney J. K., Boettcher S. W. (2014). J. Am. Chem. Soc..

[cit36] Li N., Hadt R. G., Hayes D., Chen L. X., Nocera D. G. (2021). Nat. Commun..

[cit37] Wang Y. H., Wang X. T., Ze H., Zhang X. G., Radjenovic P. M., Zhang Y. J., Dong J. C., Tian Z. Q., Li J. F. (2021). Angew. Chem., Int. Ed. Engl..

[cit38] Li C. Y., Dong J. C., Jin X., Chen S., Panneerselvam R., Rudnev A. V., Yang Z. L., Li J. F., Wandlowski T., Tian Z. Q. (2015). J. Am. Chem. Soc..

[cit39] Li W. Q., Zhou R. Y., Wang X. T., Hu L. Y., Chen X., Guan P. C., Zhang X. G., Zhang H., Dong J. C., Tian Z. Q., Li J. F. (2021). J. Catal..

[cit40] Wang X., Li T. (2020). Spectrochim. Acta, Part A.

[cit41] Zhao R., Yue X., Li Q., Fu G., Lee J. M., Huang S. (2021). Small.

[cit42] Liu E., Li J., Jiao L., Doan H. T. T., Liu Z., Zhao Z., Huang Y., Abraham K. M., Mukerjee S., Jia Q. (2019). J. Am. Chem. Soc..

[cit43] Grimaud A., Diaz-Morales O., Han B., Hong W. T., Lee Y. L., Giordano L., Stoerzinger K. A., Koper M. T. M., Shao-Horn Y. (2017). Nat. Chem..

[cit44] Xiao H., Shin H., Goddard W. A. (2018). Proc. Natl. Acad. Sci. U. S. A..

[cit45] Li J. F., Tian X. D., Li S. B., Anema J. R., Yang Z. L., Ding Y., Wu Y. F., Zeng Y. M., Chen Q. Z., Ren B., Wang Z. L., Tian Z. Q. (2013). Nat. Protoc..

[cit46] Kresse G., Furthmüller J. (1996). Comput. Mater. Sci..

[cit47] Kresse G., Hafner J. (1994). Phys. Rev. B: Condens. Matter Mater. Phys..

[cit48] Perdew J. P., Burke K., Ernzerhof M. (1996). Phys. Rev. Lett..

[cit49] Kresse G., Furthmuller J. (1996). Phys. Rev. B: Condens. Matter Mater. Phys..

[cit50] Friebel D., Louie M. W., Bajdich M., Sanwald K. E., Cai Y., Wise A. M., Cheng M. J., Sokaras D., Weng T. C., Alonso-Mori R., Davis R. C., Bargar J. R., Norskov J. K., Nilsson A., Bell A. T. (2015). J. Am. Chem. Soc..

[cit51] Grimme S., Antony J., Ehrlich S., Krieg H. (2010). J. Chem. Phys..

[cit52] Grimme S., Ehrlich S., Goerigk L. (2011). J. Comput. Chem..

[cit53] Monkhorst H. J., Pack J. D. (1976). Phys. Rev. B.

