# Supplementary material for: In situ Raman spectroscopy reveals the structure evolution and lattice oxygen reaction pathway induced by the crystalline–amorphous heterojunction for water oxidation
Source: Chem Sci. 2022 Apr 22;13(19):5639–49. doi: 10.1039/d2sc01043g (PMC9116351; doi:10.1039/d2sc01043g)
Supplement: SC-013-D2SC01043G-s001 [file SC-013-D2SC01043G-s001.pdf]

Electronic Supplementary Material (ESI) for Chemical Science.
This journal is © The Royal Society of Chemistry 2022

In situ Raman spectroscopy reveals the structure evolution and lattice oxygen reaction pathway induced by the crystalline-amorphous heterojunction for water oxidation

Jianing Dong,[a, †] Zhengxin Qian,[a, †] Pan Xu,[a] Mu-Fei Yue,[a] Ru-Yu Zhou,[a] Yanjie Wang,[a] Zi-Ang Nan,[a] Siying Huang,[a] Quanfeng Dong,[a] Jian-Feng Li,[a, b] Feng Ru Fan,[a,*] Zhong-Qun Tian[a]

[a]State Key Laboratory of Physical Chemistry of Solid Surfaces, College of Chemistry and Chemical Engineering, Innovation Laboratory for Sciences and Technologies of Energy Materials of Fujian Province (IKKEM), Xiamen University, Xiamen 361005, China.

[b]College of Optical and Electronic Technology, China Jiliang University, Hangzhou, China.

*Corresponding author:

Email: frfan@xmu.edu.cn

Supplementary Discussions about the synthesis details

When a water drop touches a hot plate whose temperature is much higher than the boiling point of water, the part of the drop which comes in contact with the hot surface vapourizes immediately and the drop levitates on its own vapour. This effect is called Leidenfrost effect, was first investigated by Leidenfrost.[1] The boiling mode of a droplet is related to the collision velocity of the droplet with a high temperature solid surface. The velocity of droplet collision is usually characterized by the Weber number (We), which can be can be interpreted as the ratio of kinetic energy to the surface tension of the droplet:[2]

We = ρv2d/γ

In this formula, ρ, d, v, γ represents the density, diameter, impact velocity and surface tension of the droplet, respectively. When the We is close to 0, the boiling mode of droplets is mainly analyzed from the perspective of thermodynamics, which is called thermodynamic boiling mode (Figure S1a).[3] As shown in Figure R5, the boiling of droplets on solid surfaces is generally divided into three modes: nucleate boiling, transition boiling, and film boiling, which is changed with increasing temperature. When the We is large, vapor layer may be destroyed due to high impact velocity. Therefore, the synergistic relationship between surface temperature and We needs to be considered.

The spray pattern of electrospray is closely related to the conductivity and the applied voltage of the liquid, and the polarity of the voltage can provide different chemical reaction environment. Based on our previous report, in this system, the

voltage of -2.5 ± 0.5 kV is applied to obtain the uniform spray of cone-jet mode. Simultaneously, Once the potential of electrospray is determined, the diameter of droplet can be estimated. Moreover, Sun and co-workers reported that the Leidenfrost temperature needed for droplets on carbon fiber substrates at different Weber numbers.[2,4] In this work, we calculated the Weber number of droplets based on previous report and combined with high thermal conductivity of graphite phase carbon paper. The substrate of the heating plate is set at 290 °C to avoid the air layer weakening the effect of heat transfer.

We first discussed the effects of spray needle diameter and precursor concentration on the size of nanoparticles. The spray time was initially set to 1min. Firstly, we studied the influence of the diameter of the spray capillary on the nucleation size of the particles, the diameter of spray needle (50/75/100 μm) did not affect the particle size changes (Figure S2), possibly because of the small droplet size change. Then, the concentration effect was investigated with 1 min spraying time (Figure S3). When the precursor concentration of solution is 0.01 M, the droplets will produce large particles size about 5 nm. As the concentration increases, the size of nanoparticles decreases, forming ultrafine nanoparticles (~3 nm). However, whether the precursor concentration is high or low, the nanocrystals produced by suspension droplets exhibit a highly integrated monodisperse property compared with the traditional spray pyrolysis technology (Figure S1b). At the same time, the scale is reduced from submicron and micron to nanometer. Further, we increased the spray time by 10 min, the electrocatalysts accidentally formed the nanowire array (Figure 1b). To further explain

this phenomenon, the spray time is set at 2 min and 5 min (Figure S4): we found that the nanoparticles will accumulate and grow along the axial direction. In a recent work, three dimensional nano-printing is realized by electric field-assisted charged aerosol and metallic nano-brushes made using ambient droplet electrosprays.[5, 6] Based on this concept, to prove the guiding effect of electric field, we further characterized the structure of nanowires, as shown in (Figure 1c-d) It is shown that the nanowires are connected by multiple particles. Meanwhile, SAED images show the existence of polycrystalline microparticles (Figure 1f). Both have proved that the electric field induces the assembly of nanoparticles, and the nanowire arrays are formed with extending the spray time. Generally, different element ratios will produce different catalyst morphologies. We also explored the morphologies of catalysts with different element ratios. (Figure S5) Other electrocatalysts with different Ni : Fe ratios also present nanowire arrays. Therefore, the formation of nanowire structures is more of a self-assembly behavior rather than crystallization. Based on the above analysis, we proposed the potential mechanism of catalyst growth: (1) The micro-droplets produced by electrospray firstly have a Coulomb explosion, producing droplets with different sizes. (2) When these droplets with different sizes contact the overheated substrate, the formation of the suspended droplets will have two fates under the action of Leidenfrost: (1) When the droplet size is larger than the critical size R_c, the droplet will explode and produce small droplets; (2) When the droplet size is smaller than the critical size R_c, the droplet evaporates and crystallizes to form nanocrystals. These two effects unify the droplet size and evaporation rate, making the nucleation of micro and nano crystals at

the same saturation and crystallization rate, resulting in monodisperse nanoparticles. With the prolongation of the spray time, nanocrystals are further assembled to form uniform nanowire arrays assisted by the tip electric field. The whole preparation process was shown in Figure 1a.

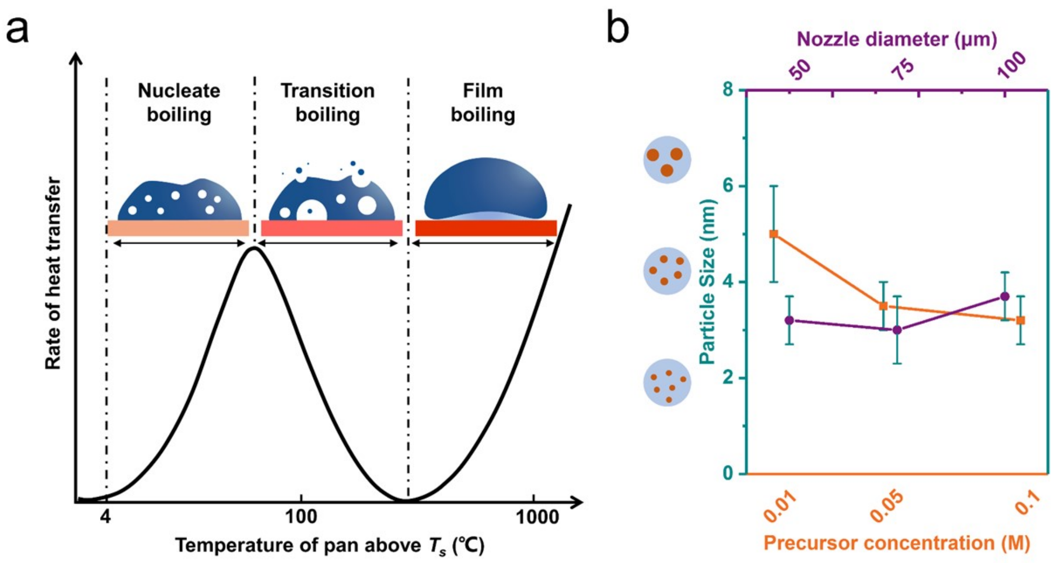

Figure S1. (a) The curve of water boiling at different temperatures (at 1 atm). (b) Particle size of a-NiO_x/α-Fe_2O_3 prepared via different nozzle diameters and precursor concentrations.

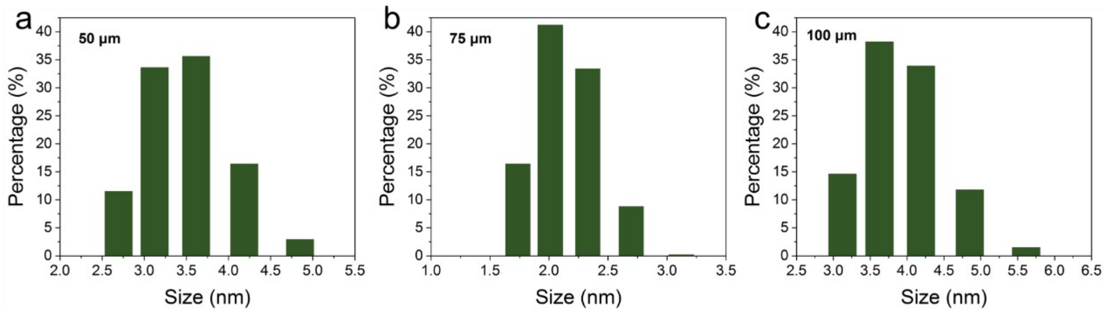

Figure S2. Size distribution of a-NiO_x/α-Fe_2O_3 prepared via different nozzle diameters (50 μm, 75 μm and 100 μm).

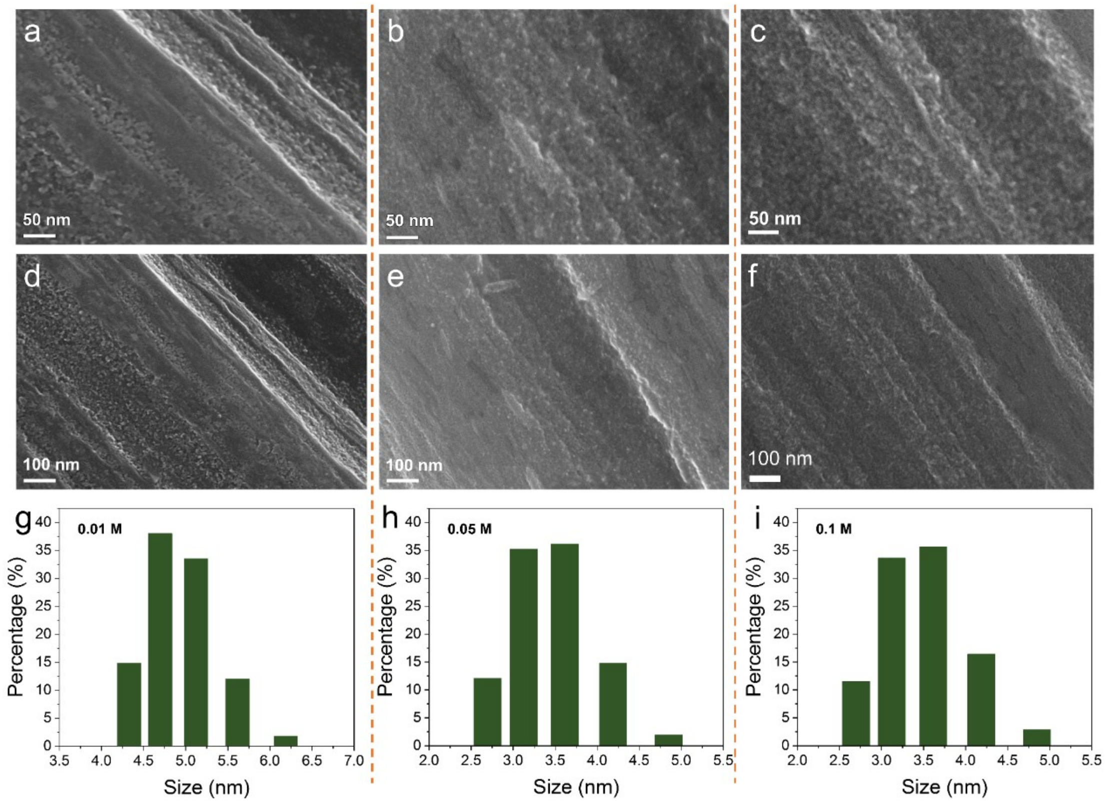

Figure S3. SEM images and size distribution of a-NiO_x/α-Fe_2O_3 prepared via different precursor concentrations with 1 min spraying time. (0.01 M, 0.05 M and 0.1 M).

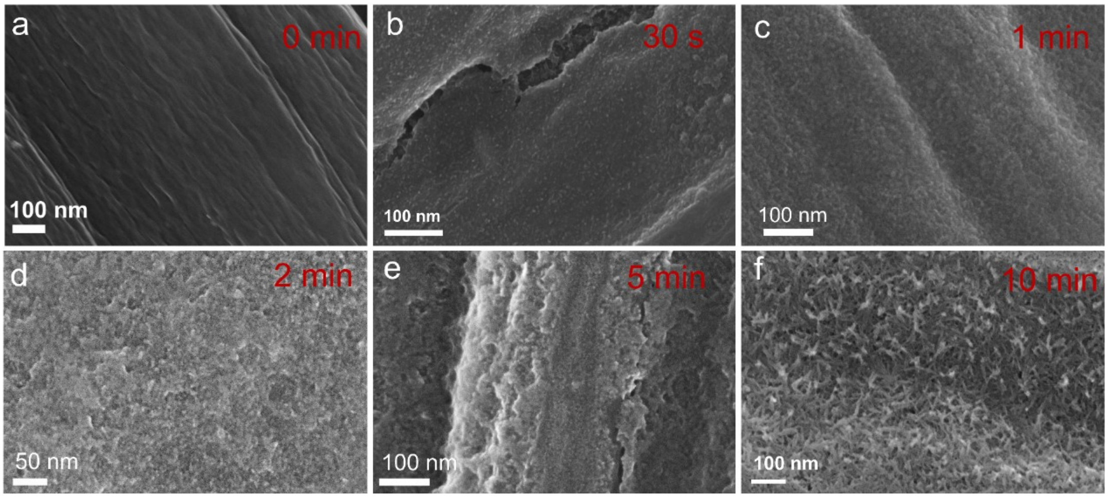

Figure S4. SEM images a-NiO_x/α-Fe_2O_3 prepared via different spraying time.

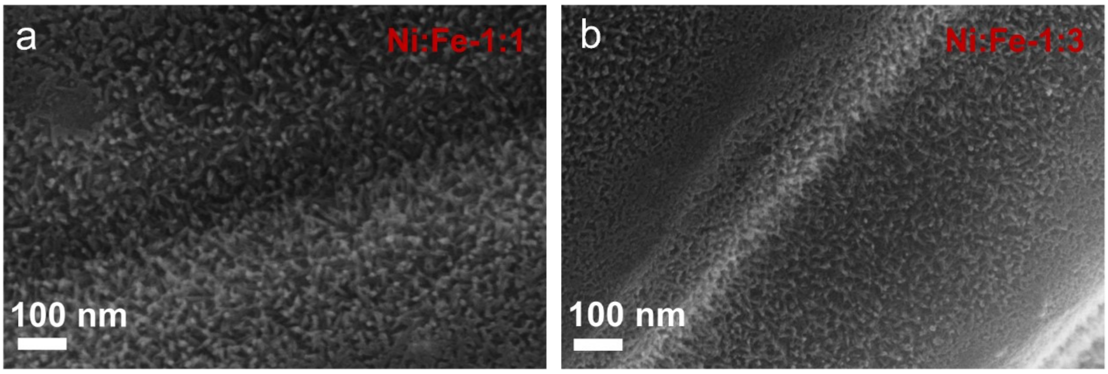

Figure S5. SEM images a-NiO_x/α-Fe_2O_3 with different Ni : Fe ratio.

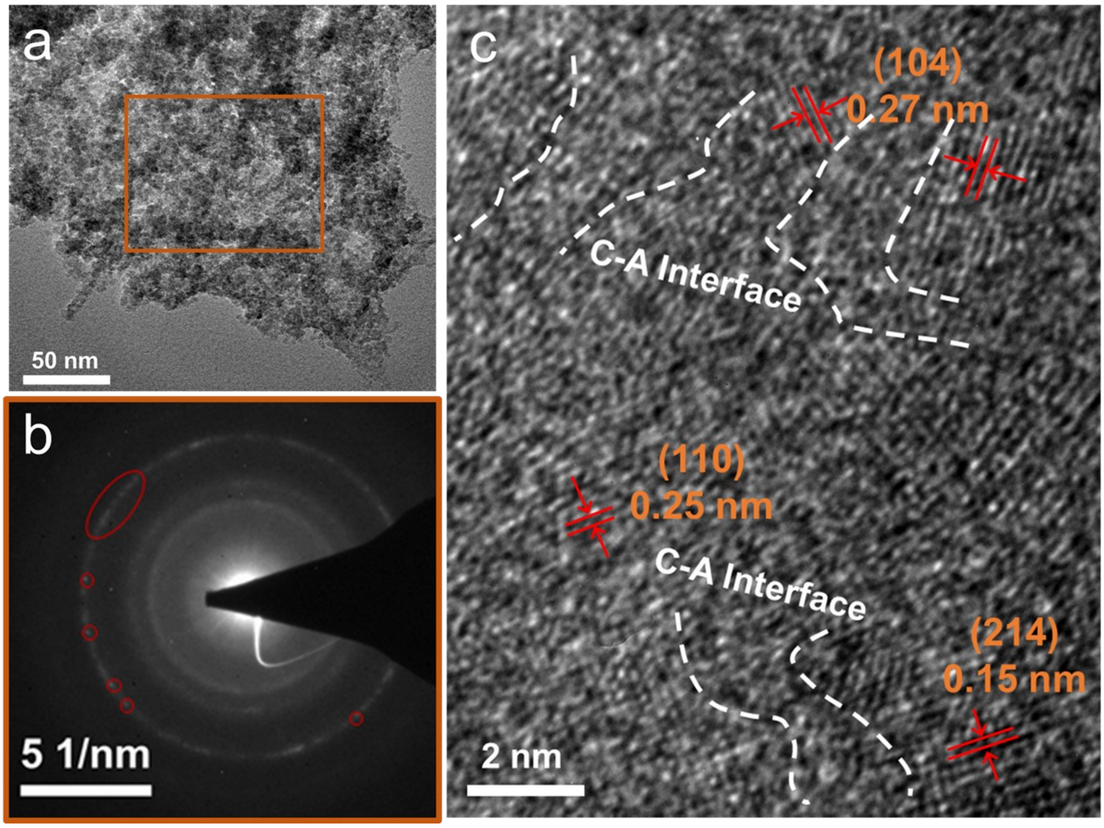

Figure S6. (a) TEM image and SAED pattern (b) of a-NiO_x/α-Fe_2O_3. (c) HRTEM image of a-NiO_x/α-Fe_2O_3, which shows obvious c-a interface.

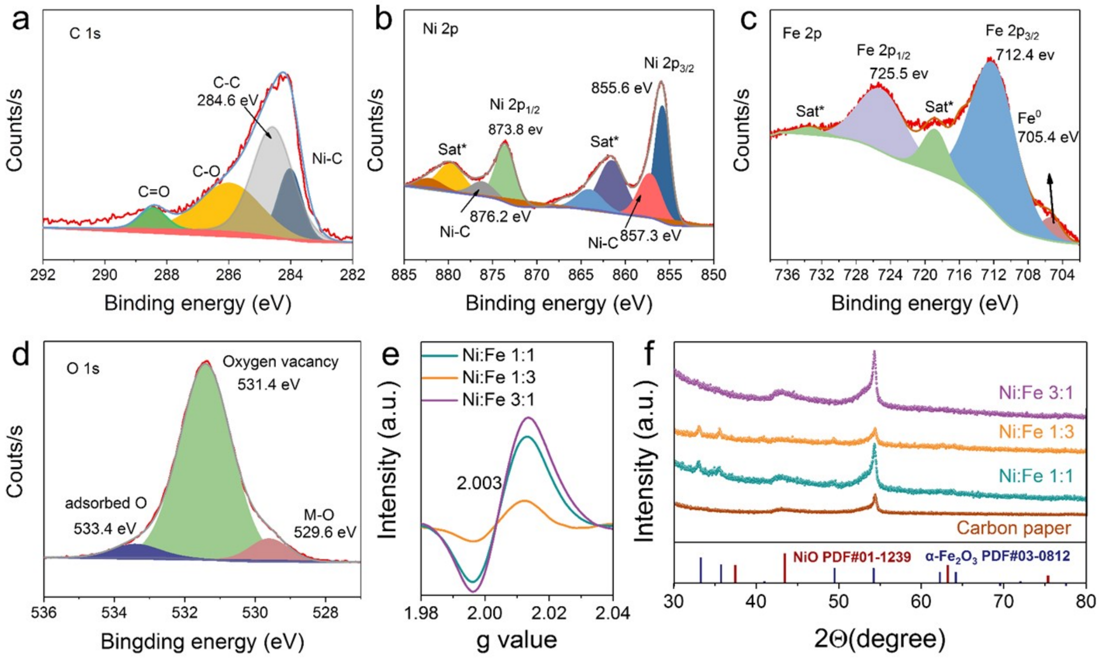

Figure S7. High-resolution XPS spectra of C 1s (a), Ni 2p (b), Fe 2p (c) and O 1s (d) of a-NiO_x/α-Fe_2O_3. EPR spectra (e) and XRD parttern (f) of a-NiO_x/α-Fe_2O_3 with different Ni :Fe ratio.

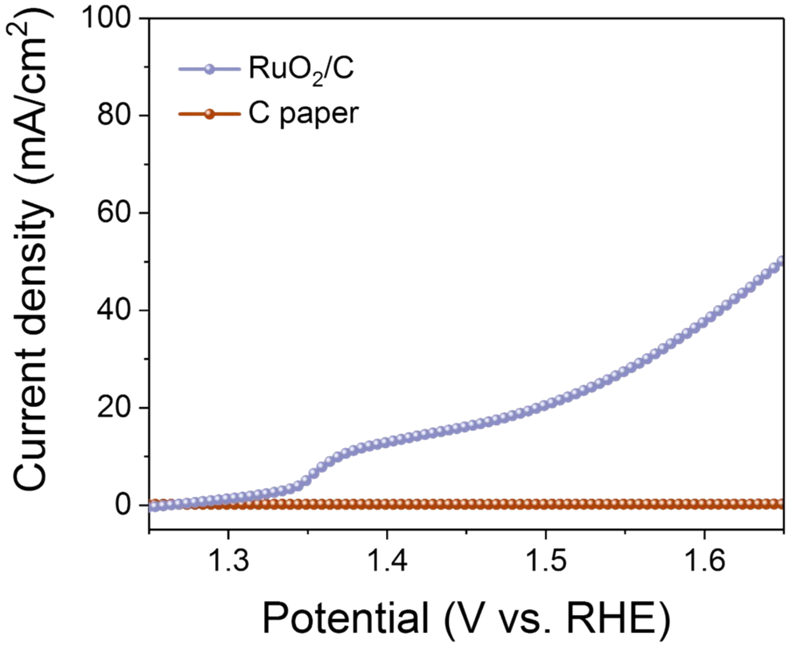

Figure S8. LSV curves for OER measured on the commercial RuO_2 on carbon paper and pristine carbon paper.

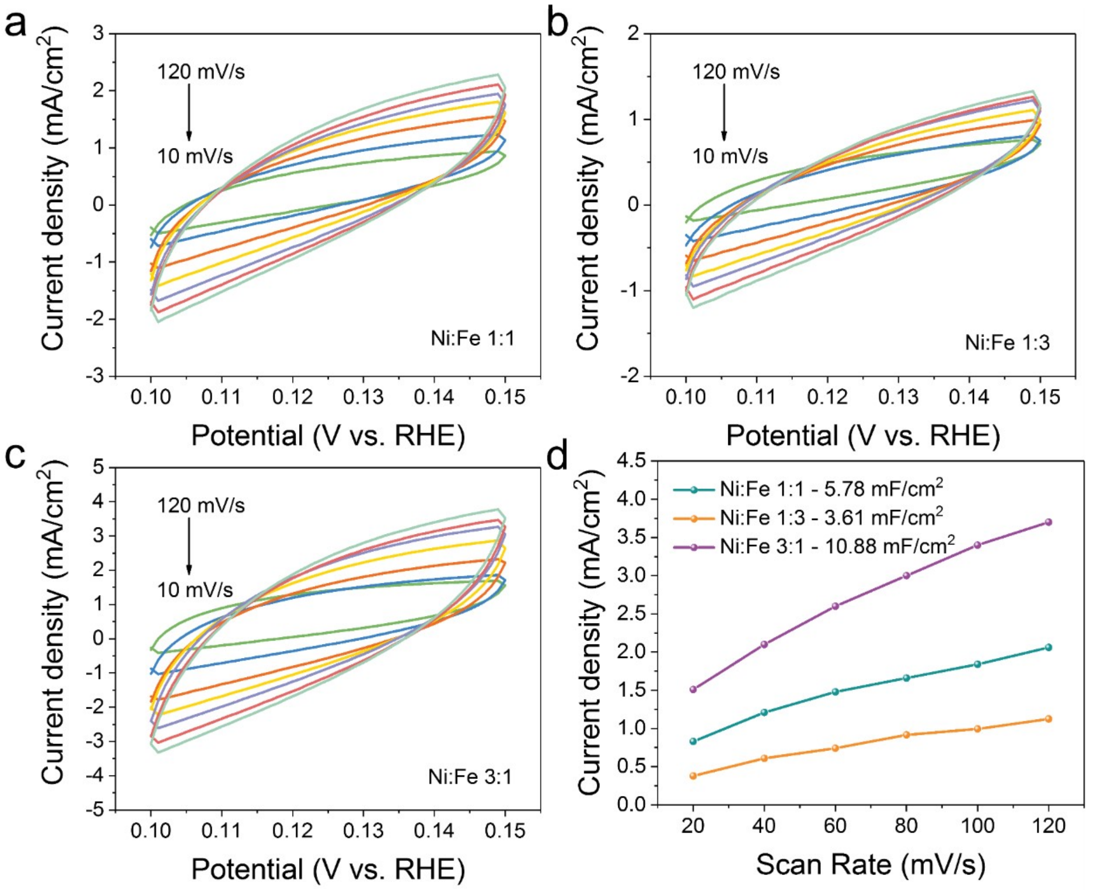

Figure S9. The determination of C_{dl} for various catalysts. (a-c) CV scanning curves of (a) Ni_1Fe_1 (b) Ni_1Fe_3, and (c) Ni_3Fe_1 catalysts in 1M KOH solution at different scan rates in the non-Faradaic potential region (0.1 - 0.15 V versus RHE). (d) Capacitive current density differences ($\Delta J = (J_+ - J_-)$) at 0.12 V versus RHE as a function of scan rate. The linear slope is double equivalent to C_{dl}.

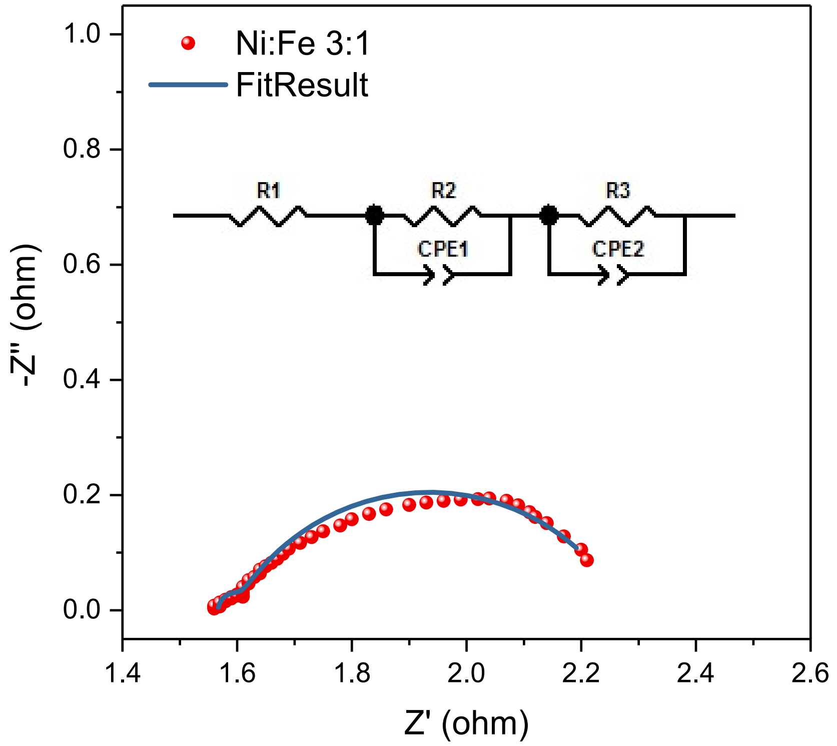

Figure S10. The EIS fitting result of Ni_3Fe_1. The red ball represents the EIS test data. The blue curve is the fitting result of Ni_3Fe_1. The inset is the equivalent circuit diagram with using CPE as capacitor models.

Table S1. The elements values and errors in the equivalent circuit.

Element	Value	Error %
R1 (Rs)	1.566	0.21711
R2	0.68552	2.0571
CPE1-T	0.4569	2.6347
CPE1-P	0.68571	2.0668
R3	0.026275	5.9817
CPE2-T	0.013068	19.455
CPE2-P	1.097	2.3727

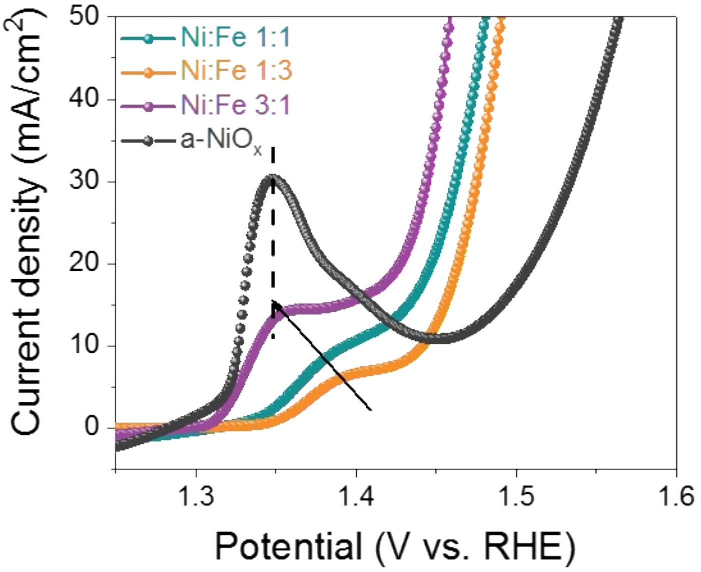

Figure S11. LSV measurement curves of different electrocatalysts. Compare with the LSV curves of a-NiO$_x$ to distinguish between electron effects and concentration effects.

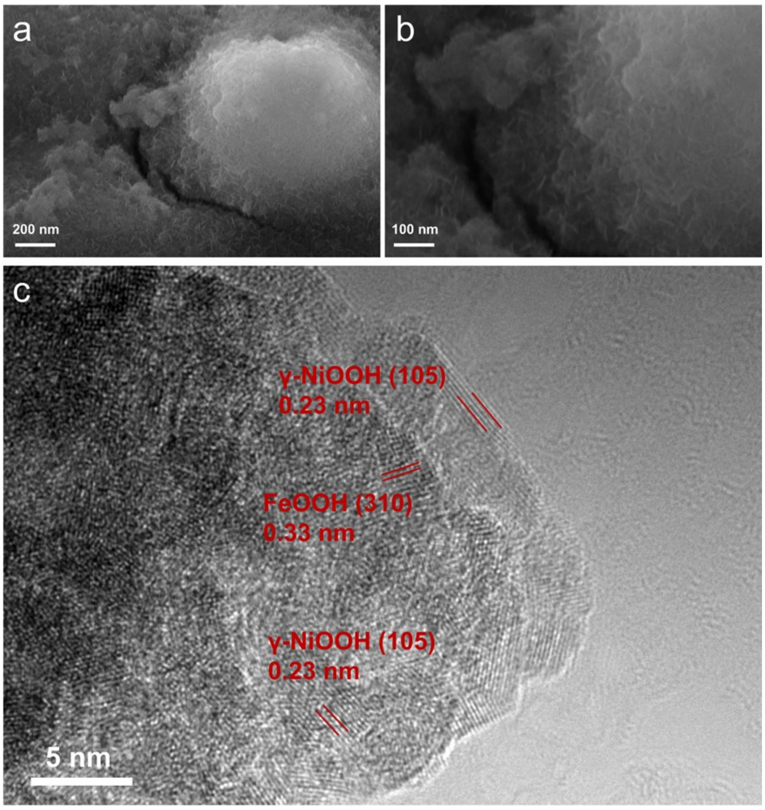

Figure S12. (a) - (b) SEM images of OER-activated electrocatalysts. (b) HRTEM imaged of OER-activated electrocatalysts, which is the γ-NiOOH/FeOOH componests.

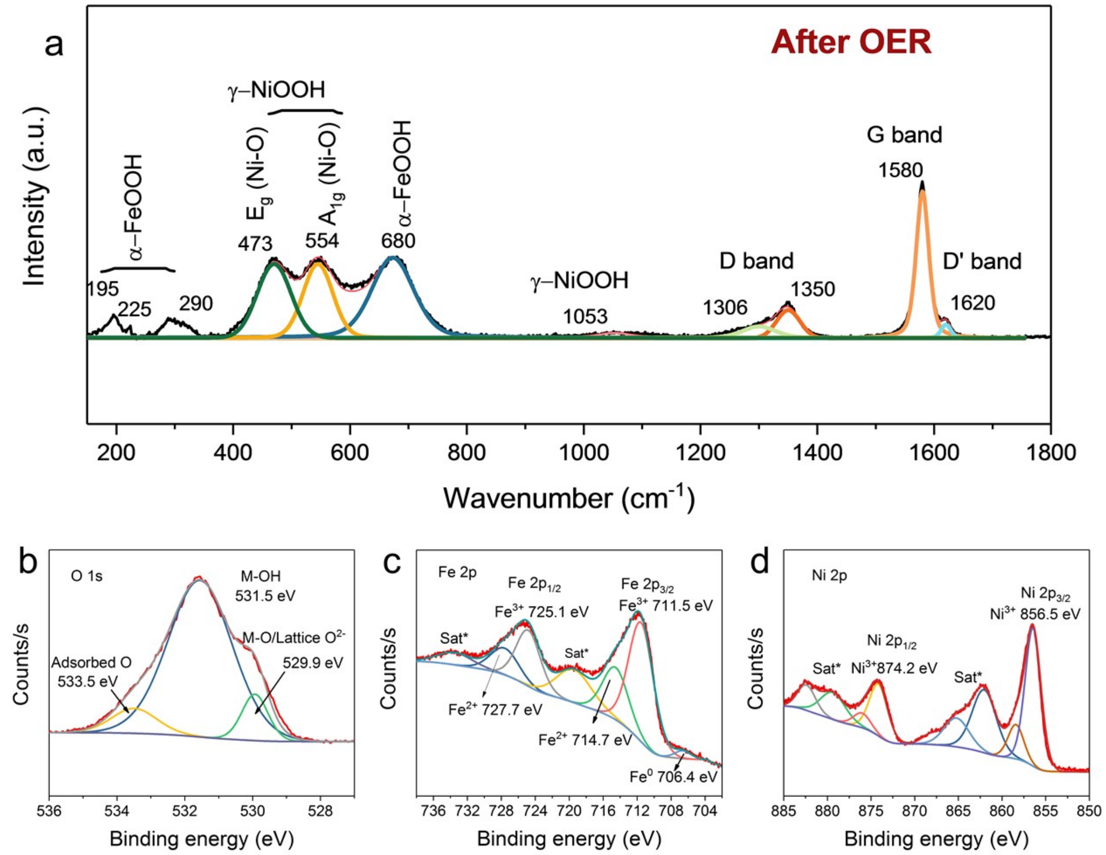

Figure S13. (a) Raman spectra of OER-activated electrocatalysts. High-resolution XPS spectra of O 1s (b), Fe 2p (c) and Ni 2p (d) of OER-activated electrocatalysts.

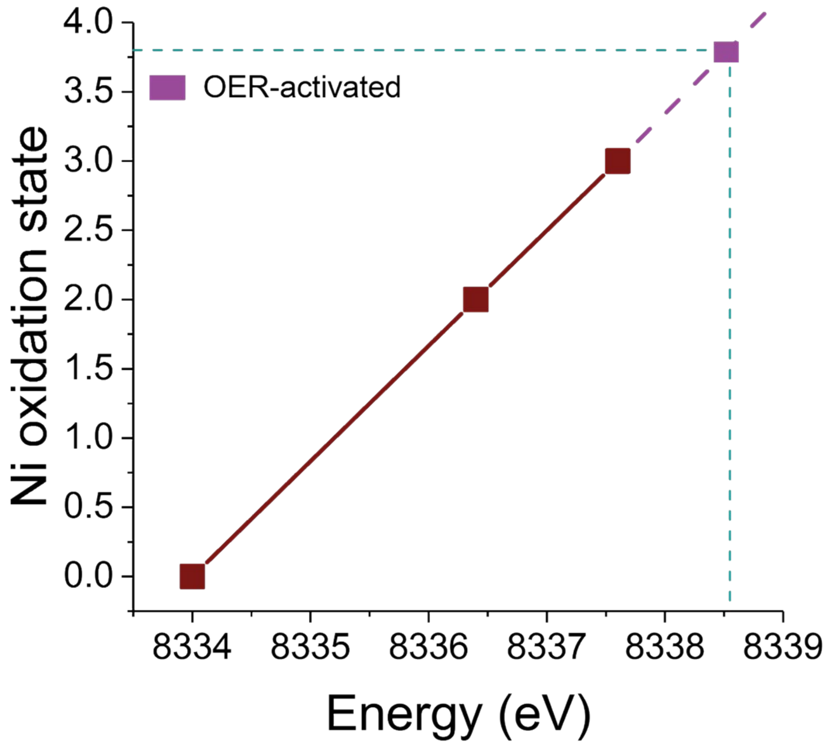

Figure S14. Ni oxidation state as a function of the XANES energy value.

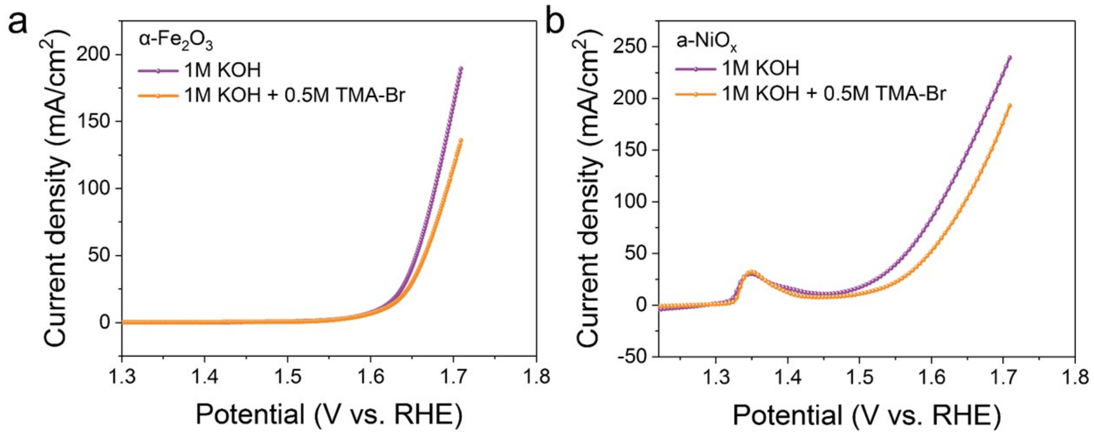

Figure S15. LSV curves of α-Fe_2O_3 (a) and a-NiO_x (b) in 1 M KOH and 1 M KOH with TMA^+, respectively.

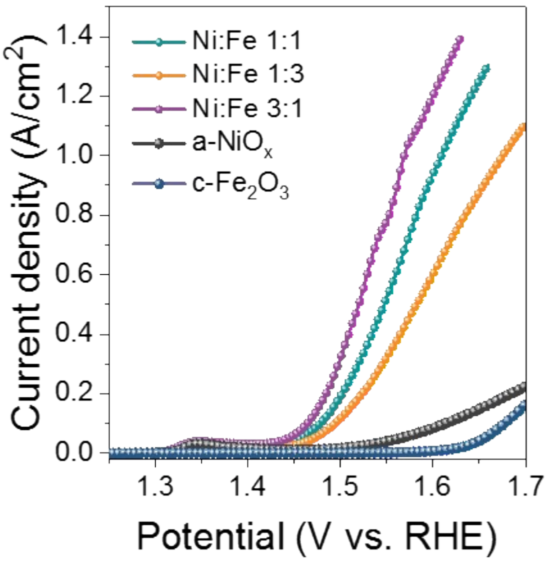

Figure S16. The LSV curves of different electrocatalysts. Compared with a-NiO_x and c-Fe_2O_3 prepared using the same methods as NiFe based electrocatalysts to exclude the influence of Fe impurities.

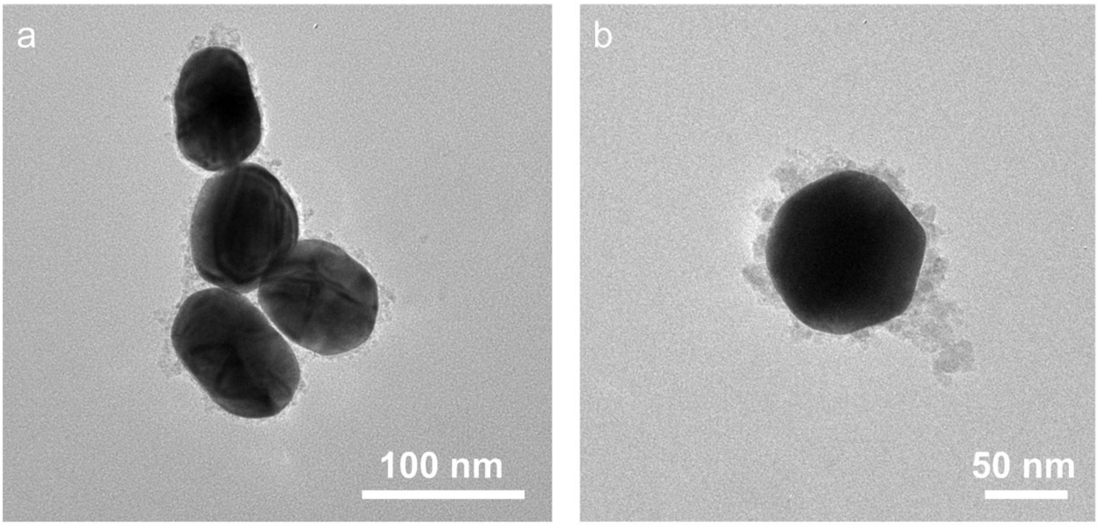

Figure S17. TEM images of NiO_x-SAT (a) and NiO_x/Fe_2O_3-SAT (b).

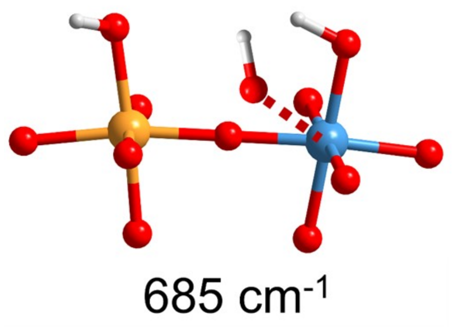

Figure S18 Optimized adsorption configuration and vibration frequency of *OH absorbed on the Ni site.

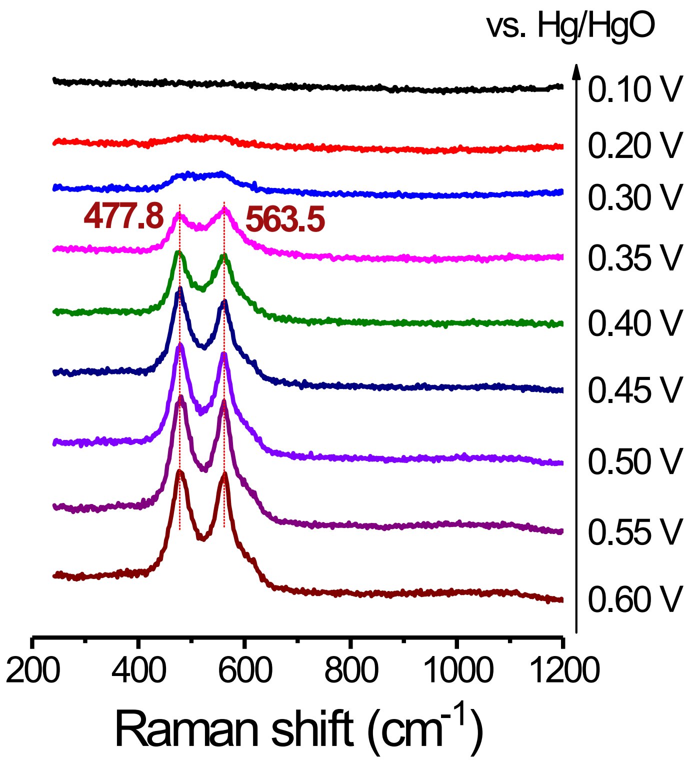

Figure S19. In-situ SHINERS of OER on a-NiO_x in 1 M KOH dissolved in H_2O.

Table S2. Comprehensive comparison of the OER performance between the a-NiO_x/α-Fe_2O_3 catalyst and other recently reported OER electrocatalysts. Here η_{100} represent the overpotentials required to achieve current densities of 100 mA /cm^2.

**Electrocatalysts**	**The largest test current density**	**η_{100} (mV)**	**Current density for stability test**	**References**	**Electrolyte**
S-(NiFe)OOH[7]	1000 mA	281 mV	500 mA	Energy Environ. Sci., 2020, 13, 3439-3446	1M KOH
FeOOH(Se)[8]	550 mA	279 mV	100 mA	J. Am. Chem. Soc., 2019, 141, 17, 7005-7013	1M KOH
NiFeRu-LDH[9]	420 mA	260 mV*	10 mA	Adv. Mater., 2018, 30, 1706279	1M KOH
Ru/D-NiFeLDH[10]	350 mA	218 mV	100 mA	Nat. Commun., 2021, 12, 4587.	1M KOH
hcp-NiFe@NC[11]	400 mA	263 mV	20 mA	Angew. Chem. Int. Ed., 2019, 58, 6099-6103	1M KOH
NiFeSe-dO[12]	100 mA	247 mV	100 mA	Angew. Chem. Int. Ed., 2019, 58, 6099-6103	1M KOH
NiMoFeO@NC[13]	400 mA*	290 mV	100 mA	Matter 2020, 3 (6), 2124-2137	1M KOH
FeCoCrNi[14]	410 mA*	250 mV*	10 mA	Nat. Commun., 11, 4066 (2020)	1M KOH
Cu@NiFe LDH[15]	1000 mA	281 mV	100 mA	Energy Environ. Sci., 2017, 10, 1820-1827	1M KOH
NiFe LDH/NiS[16]	1000 mA	386 mV	400 mA	Adv. Energy. Mater., 2021, 2102353.	1M KOH
cMOF/LDH[17]	370 mA*	227 mV	300mA	Adv. Mater., 2021, 33, 2006351	1M KOH
a-NiO_x/c-Fe_2O_3	1400 mA	230 mV	1200mA	**This work**	1M KOH

* The value is calculated from the curves shown in the literature.

Table S3. The uncompensated resistance changed for the different KOH concentrations.

Electrolyte	pH value	Resistance (ohm)
KOH	13.7	1.6
KOH	13.2	5.5
KOH	12.7	13.3
KOH	12.2	28.6

References:

1. Leidenfrost, J. G. D*e Aquae Communis Nonnullis Qualitatibus Tractatus* (Johann Straube, Duisburg, Germany, 1756).
2. H. Nair, H. J. Staat, T. Tran, A. van Houselt, A. Prosperetti, D. Lohse and C. Sun, *Soft Matter*, 2014, **10**, 2102-2109.
3. G. Liang, I. Mudawa, *Int. J. Heat Mass Tran.,* 2017, **106**, 103-126.
4. S. Lyu, V. Mathai, Y. Wang, B. Sobac, P. Colinet, D. Lohse and C. Sun, *Sci Adv*, 2019, **5**, eaav8081.
5. W. Jung, Y. H. Jung, P. V. Pikhitsa, J. Feng, Y. Yang, M. Kim, H. Y. Tsai, T. Tanaka, J. Shin, K. Y. Kim, H. Choi, J. Rho and M. Choi, *Nature*, 2021, **592**, 54-59.
6. D. Sarkar, M. K. Mahitha, A. Som, A. Li, M. Wleklinski, R. G. Cooks and T. Pradeep, *Adv. Mater.*, 2016, **28**, 2223-2228.
7. L. Yu, L. Wu, B. McElhenny, S. Song, D. Luo, F. Zhang, Y. Yu, S. Chen and Z. Ren, *Energy & Environmental Science*, 2020, DOI: 10.1039/d0ee00921k.
8. S. Niu, W. J. Jiang, Z. Wei, T. Tang, J. Ma, J. S. Hu and L. J. Wan, *J. Am. Chem. Soc.*, 2019, **141**, 7005-7013.
9. G. Chen, T. Wang, J. Zhang, P. Liu, H. Sun, X. Zhuang, M. Chen and X. Feng, *Adv. Mater.*, 2018, **30**.
10. P. Zhai, M. Xia, Y. Wu, G. Zhang, J. Gao, B. Zhang, S. Cao, Y. Zhang, Z. Li, Z. Fan, C. Wang, X. Zhang, J. T. Miller, L. Sun and J. Hou, *Nat. Commun.*, 2021, **12**, 4587.
11. C. Wang, H. Yang, Y. Zhang and Q. Wang, *Angew. Chem. Int. Ed. Engl.*, 2019, **58**, 6099-6103.
12. A. Peugeot, C. E. Creissen, D. Karapinar, H. N. Tran, M. Schreiber and M. Fontecave, *Joule*, 2021, **5**, 1281-1300.
13. Y. Wang, Y. Zhu, S. Zhao, S. She, F. Zhang, Y. Chen, T. Williams, T. Gengenbach, L. Zu, H. Mao, W. Zhou, Z. Shao, H. Wang, J. Tang, D. Zhao and C. Selomulya, *Matter*, 2020, **3**, 2124-2137.
14. N. Zhang, X. Feng, D. Rao, X. Deng, L. Cai, B. Qiu, R. Long, Y. Xiong, Y. Lu and Y. Chai, *Nat. Commun.*, 2020, **11**, 4066.
15. L. Yu, H. Zhou, J. Sun, F. Qin, F. Yu, J. Bao, Y. Yu, S. Chen and Z. Ren, *Energy & Environmental Science*, 2017, **10**, 1820-1827.

16. Q. Wen, K. Yang, D. Huang, G. Cheng, X. Ai, Y. Liu, J. Fang, H. Li, L. Yu and T. Zhai, *Advanced Energy Materials*, 2021, DOI: 10.1002/aenm.202102353.
17. Y. Wang, L. Yan, K. Dastafkan, C. Zhao, X. Zhao, Y. Xue, J. Huo, S. Li and Q. Zhai, *Adv. Mater.*, 2021, **33**, e2006351.
